# Incidence and mortality of lung cancer: global trends and association with socioeconomic status

**DOI:** 10.1038/s41598-017-14513-7

**Published:** 2017-10-30

**Authors:** Martin C. S. Wong, Xiang Qian Lao, Kin-Fai Ho, William B. Goggins, Shelly L. A. Tse

**Affiliations:** 1 Division of Family Medicine and Primary Healthcare, School of Public Health and Primary Care, Faculty of Medicine, Chinese University of Hong Kong, Hong Kong, China; 2 Family Medicine and Primary Health Care, Hospital Authority, Hong Kong, China; 3 Division of Occupational and Environmental Health, School of Public Health and Primary Care, Faculty of Medicine, Chinese University of Hong Kong, Hong Kong, China; 4 Division of Biostatistics, School of Public Health and Primary Care, Faculty of Medicine, The Chinese University of Hong Kong, Hong Kong, China

## Abstract

We examined the correlation between lung cancer incidence/mortality and country-specific socioeconomic development, and evaluated its most recent global trends. We retrieved its age-standardized incidence rates from the GLOBOCAN database, and temporal patterns were assessed from global databases. We employed simple linear regression analysis to evaluate their correlations with Human Development Index (HDI) and Gross Domestic Product (GDP) per capita. The average annual percent changes (AAPC) of the trends were evaluated from join-point regression analysis. Country-specific HDI was strongly correlated with age-standardized incidence (r = 0.70) and mortality (r = 0.67), and to a lesser extent GDP (r = 0.24 to 0.55). Among men, 22 and 30 (out of 38 and 36) countries showed declining incidence and mortality trends, respectively; whilst among women, 19 and 16 countries showed increasing incidence and mortality trends, respectively. Among men, the AAPCs ranged from −2.8 to −0.6 (incidence) and −3.6 to −1.1 (mortality) in countries with declining trend, whereas among women the AAPC range was 0.4 to 8.9 (incidence) and 1 to 4.4 (mortality) in countries with increasing trend. Among women, Brazil, Spain and Cyprus had the greatest incidence increase, and all countries in Western, Southern and Eastern Europe reported increasing mortality. These findings highlighted the need for targeted preventive measures.

## Introduction

Worldwide, lung cancer is the most common malignancy and the most common cause of cancer deaths in the past few decades^1^. In 2012, a total of 1.8 million new cases were estimated, accounting for 12.9% of all new cancer diagnoses. According to the Global Burden of Disease study 2020^2^, the healthcare burden and costs attributed to lung cancer was substantial on a global scale. Its five-year survival rate (17.8%) was much lower than that of other leading cancers^3^. Owing to the high fatality rate^4^, its geographical mortality patterns closely follow those of incidence, and it remains to be an important public health issue.

Approximately 58% of all lung cancers occurred in less developed nations^5^. The major types of lung cancer include adenocarcinoma, squamous cell carcinoma, small cell and large cell carcinoma, and a previous study showed that ethnic and racial differences in relative risk of lung cancer exist for all histologic types^6^. The most important risk factor for lung cancer is tobacco smoking. Other contributory factors include environmental exposure to radon; asbestos; certain metals such as chromium, cadmium and arsenic; some organic chemicals; radiation; coal smoke; as well as indoor emission of fuel burning^7,8^. Since these risk factors are highly preventable by smoking cessation and clean air initiatives, it should be possible to reduce its incidence and consequent mortality by population-based preventive strategies^9^. Hence, it is crucial to understand its global epidemiology, particularly with respect to temporal patterns and trends.

Previous studies describing international trends of lung cancer used figures from registries in early 2000s^4,8,10–12^, focused on a single or few countries^4,13^, presented purely descriptive statistics^14^, or depended on comparisons across countries in a single calendar year^5^. There are at least two important knowledge gaps yet to be further addressed. Firstly, few studies have delineated the association between lung cancer incidence/mortality and country-specific socioeconomic development, and there has been a scarcity of research that examined how macroeconomic determinants correlate with the incidence and mortality of this cancer. Also, recent studies implied that lung cancer incidence and mortality might be rising in women but declining in men, yet the analyses were constrained to the United States^4,13^. Evaluation of temporal trends of this cancer could quantify geographical variation, identify high-risk populations, and delineate the potential for preventive strategies. These epidemiological data could also be linked to the future prospects of cancer prevention and possibly screening strategies for policy-makers.

This study aimed to analyze its global patterns and temporal trends based on data from high quality cancer registries. We tested the *a priori* hypothesis that the incidence and mortality rates of lung cancer were positively correlated with higher human development and productivity across different countries. We also sought to examine whether there was gender disparity in the temporal trends of this cancer among all countries where data were available.

## Methods

### Data Source

This study has been approved by the Survey and Behavioural Research Ethics Committee of the Chinese University of Hong Kong. As this study used routinely collected anonymised electronic data consent was not required. All methods were performed in accordance with the relevant guidelines and regulations, and there were no publication of identifying information. The incidence and mortality estimates for cancer of the trachea, bronchus and lung (ICD-10 C33-34) were retrieved from the GLOBOCAN database for all countries in the world for 2012^1^, as this is the calendar year where the most updated incidence and mortality figures could be retrieved. We made reference to a recent analysis of epidemiological data on prostate and colorectal cancer^15–18^, and used similar methodology for lung cancer. We obtained data on the Human Development Index (HDI) and Gross Domestic Product (GPD) for each country in 2012 from the United Nations Human Development Report^19^. To examine time trends, information was retrieved from different sources where at least 15 consecutive years of data could be obtained. For incidence figures, we extracted high-quality national population-based cancer registries from the Cancer Incidence in Five Continents (CI5) series Volumes I–X^20^. To include incidence data for more recent years, we also utilized publicly available information from the National Institute of Health (NIH) of the United States^3^, European countries^21–23^, Australia^24^ and New Zealand^25^. The incidence data for lung cancer were allocated into different categories according to the International Classification of Diseases 10^th^ revision (ICD-10 C33-34), except for the NIH of the US where the ICD codes of C340-C343 and C348-C349 were used to retrieve cancer of the lung and bronchus.

For analysis of mortality, we used the WHO mortality data series where data quality attained criteria of medium level or above^26^, which resulted in data with extensive coverage as well as high accuracy and completeness. Death certificates were the primary data source, covering around 30% of the world population, and were compiled by the International Agency for Research on Cancer (IARC) as part of the WHO mortality database. We adopted age-standardized rates (ASRs) using the world standard population^27^. Similar to the IARC, we defined more developed countries as all regions of Europe plus Northern America, Australia/New Zealand and Japan, and less developed regions as all regions of Africa, Asia (excluding Japan), Latin America and the Caribbean, Melanesia, Micronesia and Polynesia^1^. Table 1 summarizes the data source and the calendar years available where the present analyses were based on.

**Table 1:** Data source for the age-standardized incidence and mortality rates of lung cancer.

	Incidence	Mortality
Austria	EUREG (1990–2009)	WHO (1980–2014)
Croatia	CI5 (1988–2007)	WHO (1985–2013)
Czech Republic	CI5 (1983–2007)	WHO (1986–2013)
Denmark	NORDCAN (1960–2013)	NORDCAN (1960–2013)
Estonia	CI5 (1968–2007)	WHO (1981–1982, 1985–2012)
Finland	NORDCAN (1960–2013)	NORDCAN (1960–2013)
France	CI5 (1988–2007)	WHO (1979–2011)
Germany	CI5 (1970–2007)	WHO (1990–2013)
Iceland	NORDCAN (1960–2013)	NORDCAN (1960–2012)
Italy	CI5 (1993–2007)	WHO (1979–2003, 2006–2012)
Latvia	CI5 (1988–2007)	WHO (1980–2012)
Lithuania	CI5 (1978–2007)	WHO (1981–1982, 1985–2013)
Netherlands	CI5 (1989–2007)	WHO (1979–2013)
Norway	NORDCAN (1960–2013)	NORDCAN (1960–2013)
Poland	CI5 (1978–2006)	WHO (1980–1996, 1999–2013)
Slovakia	CI5 (1968–2007)	WHO (1992–2010, 2012–2014)
Slovenia	CI5 (1963–2007)	WHO (1985–2010)
Spain	CI5 (1993–2007)	WHO (1980–2013)
Sweden	NORDCAN (1960–2013)	NORDCAN (1960–2013)
Switzerland	CI5 (1993–2007)	WHO (1995–2013)
United Kingdom	CI5 (1993–2007)	WHO (1979–2013)
Australia	AIHW (1982–2012)	AIHW (1968–2013)
New Zealand	New Zealand (1960–2012)	New Zealand (1960–2012)
Bulgaria	EUREG (1993–2007)	WHO (1980–2012)
Cyprus	EUREG (1998–2007)	WHO (1999–2000, 2004–2012)
Ireland	EUREG (1994–2009)	WHO (1979–2012)
Brazil	CI5 (1988–2007)	WHO (1979–2013)
Colombia	CI5 (1983–2007)	WHO (1984–2012)
Costa Rica	CI5 (1980–2007)	WHO (1980–2013)
Ecuador	CI5 (1985–2007)	WHO (1979–2013)
Canada	CI5 (1978–2007)	WHO (1979–2011)
USA	NIH (1975–2013)	NIH (1975–2013)
USA White	NIH (1975–2013)	NIH (1975–2013)
USA Black	NIH (1975–2013)	NIH (1975–2013)
Israel	CI5 (1963–2007)	WHO (1979–2013)
Japan	CI5 (1988–2007)	WHO (1979–2013)
Philipines	CI5 (1983–2007)	WHO (1992–2003, 2008)
Singapore	CI5 (1968–2007)	WHO (1979–2014)
Thailand	CI5 (1993–2007)	WHO (1979–1987, 1990–1992, 1994–2000, 2002–2006)
China (Hong Kong)	CI5 (1993–2007)	HA (1983–2013)

n/a: not available; AIHW: Australian Cancer Incidence and Mortality Books^22^; CI5: Cancer Incidence in Five Continents V^18^; EUREG: European Union Registration^19,21^; HA: Hospital Authority, Hong Kong http://www3.ha.org.hk/cancereg/e_a1b.asp; New Zealand: the Ministry of Health of New Zealand^23^; NIH: National Institute of Cancer of the United States^3^; NORDCAN: Nordic Cancer Registries^20^; WHO: World Health Organization^24^.

### Statistical Analysis

We employed joinpoint regression analysis to examine the incidence and mortality trends^28^, using the joinpoint statistical software version 3.4. This technique fits a series of joined straight lines to ASRs^28^. Logarithmic transformation of the rates was performed with computation of the standard errors based on binomial approximation. We specified a maximum number of three joinpoints as analysis options. To determine the direction and magnitude of the recent trends, the average annual percentage change (AAPC) and its 95% confidence intervals were evaluated for the last available 10 years. The AAPC was calculated as a geometrically weighted average of the various APCs from the joinpoint regression analysis, with weights being equivalent to the length of each segment during the specified time interval^29^. The statistical significance of AAPC was ascertained comparing its magnitude with zero, and all insignificant AAPCs were regarded as having “stable trends”. The ASRs of incidence and mortality were plotted against the HDI and GDP per capita, respectively. Simple linear regression and correlation coefficients were employed to examine their associations and the goodness-of-fit. All p values < 0.05 were regarded as statistically significant. This study was approved by the Survey and Behavioral Research Ethics Committee of the Chinese University of Hong Kong.

## Results

### Incidence and mortality of lung cancer in 2012

A total of 1.8 million new cases of lung cancer and 1.6 million related deaths were reported in 2012. The ASR of incidence and mortality was higher in more developed countries than less developed ones by 1.5 to 1.4-fold in men, and by 1.8 to 1.5-fold fold in women. The ASR of lung cancer incidence varied more than 31-fold worldwide in 2012 (Supplementary Figure 1). Among men, the highest rates were found in Central and Eastern Europe (ASR 53.5 per 100,000), Eastern Asia (50.4), Micronesia (47.5) and Southern Europe (46.4), and the lowest in Western Africa (1.7), Middle Africa (2), Eastern Africa (3.8) and Central America (10.2). Among women, the highest rates were found in Northern America (ASR 33.8 per 100,000), Northern Europe (23.7), Micronesia (22.9), Australia/New Zealand (21.7) and Western Europe (20), and the lowest in Middle Africa (0.8), Western Africa (1.1), Eastern Africa (2.2) and Northern Africa (3.1).

The mortality rates varied by approximately 32-fold worldwide in 2012. In men, the highest death rates were reported in Central and Eastern Europe (47.6), Eastern Asia (44.8) and Micronesia (41.7); whilst in women, the highest mortality was also reported in Northern America (23.5), Micronesia (20.8), and Northern Europe (19.1). The lowest mortality rates were found in Western Africa (1.5), Middle Africa (1.8), and Eastern Africa (3.5) in men. These three countries also reported the lowest mortality rates among women (ASR = 1, 0.7, and 2, respectively).

Table 2 shows the ratio between the age-standardized incidence and mortality rates in all countries where data were available. Worldwide, the ratios were 1.14 in men and 1.23 in women. Countries having the highest incidence to mortality ratios in men included Australia/New Zealand (1.39), North America (1.26), Western Europe (1.25) and Southern Europe (1.19), and the ratios were the highest for women in Australia (1.45), North America (1.44) and Western Europe (1.35).

**Table 2:** Ratios of age-standardized incidence to mortality rates by world region and genders.

Countries	Men			Women		
	ASR (I)	ASR (M)	I:M	ASR (I)	ASR (M)	I:M
North America	44	34.8	1.26	33.8	23.5	1.44
Micronesia	47.5	41.7	1.14	22.9	20.8	1.10
Eastern Asia	50.4	44.8	1.13	19.2	16.2	1.19
More developed regions	44.7	36.8	1.21	19.6	14.3	1.37
Western Europe	44	35.3	1.25	20	14.8	1.35
Central and Eastern Europe	53.5	47.6	1.12	10.4	8.3	1.25
Southern Europe	46.4	39.1	1.19	12.8	10	1.28
Northern Europe	34.6	29.7	1.16	23.7	19.1	1.24
Australia/NZ	32.7	23.5	1.39	21.7	15	1.45
Polynesia	39.3	36	1.09	13.5	14.8	0.91
**World**	**34**.**2**	**30**	**1**.**14**	**13**.**6**	**11**.**1**	**1**.**23**
Western Asia	37.6	34	1.11	7.1	6.2	1.15
Less developed regions	30	27.2	1.10	11.1	9.8	1.13
South-Eastern Asia	29.6	26.6	1.11	10.5	9.4	1.12
Caribbean	25.8	23.7	1.09	13.5	12.1	1.12
Southern Africa	26.1	23.8	1.10	10.2	9.1	1.12
South America	20.8	18.4	1.13	10.7	8.9	1.20
Melanesia	14.3	13.3	1.08	5.8	5	1.16
Northern Africa	15.6	14	1.11	3.1	2.8	1.11
South-Central Asia	11.9	10.7	1.11	3.4	3.1	1.10
Central America	10.2	9	1.13	4.9	4.3	1.14
Eastern Africa	3.8	3.5	1.09	2.2	2	1.10
Middle Africa	2	1.8	1.11	0.8	0.7	1.14
Western Africa	1.7	1.5	1.13	1.1	1	1.10

ASR: Age-standardized rates; I: incidence; M: mortality.

### The correlation between incidence/mortality of lung cancer and socioeconomic development

**Figure 1:**
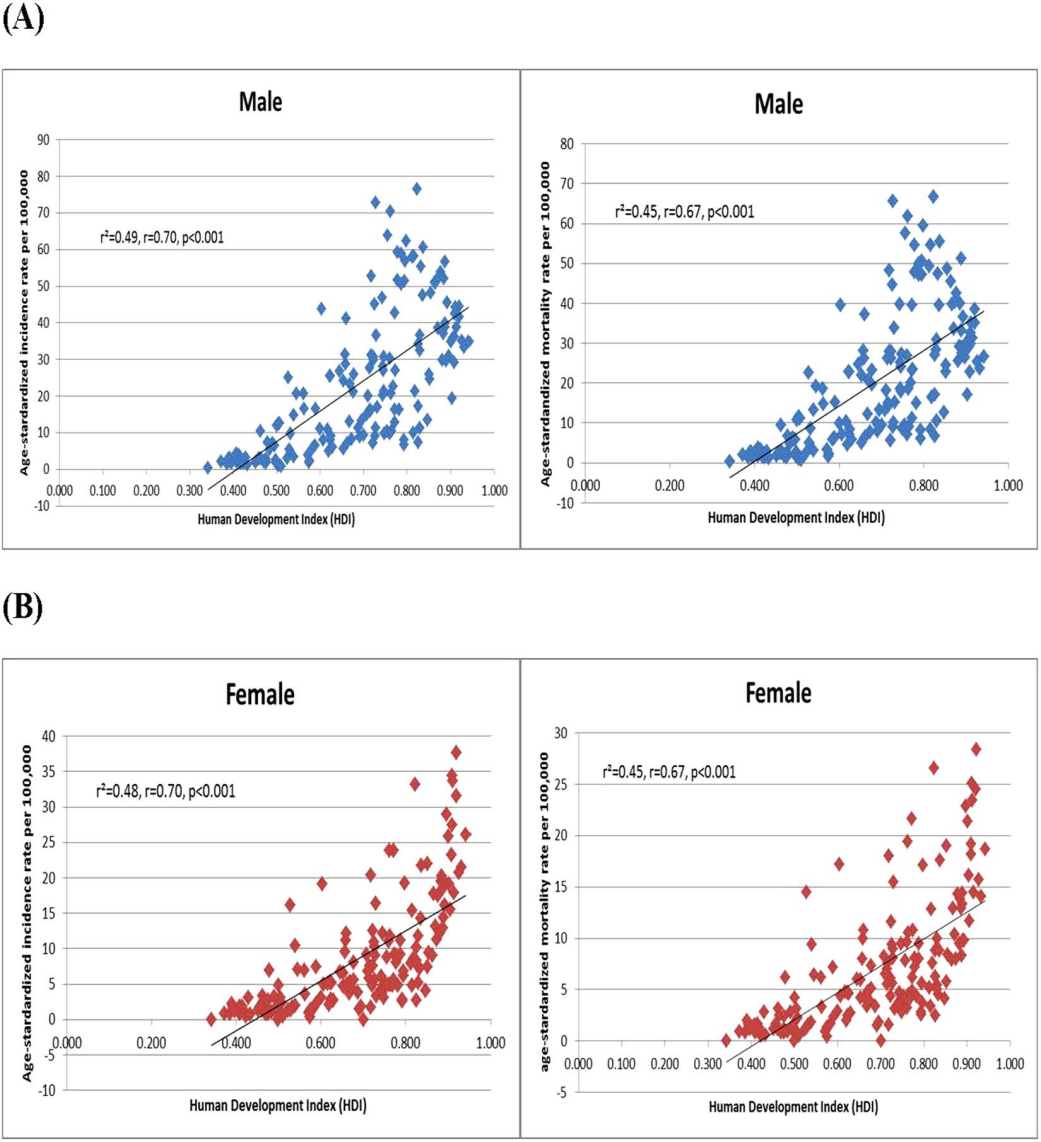
(**A**) Correlation between age-standardized lung cancer incidence (left) and mortality (right) and Human Development Index (HDI) (Male). (**B**) Correlation between age-standardized lung cancer incidence (left) and mortality (right) and Human Development Index (HDI) (Female).

**Figure 2:**
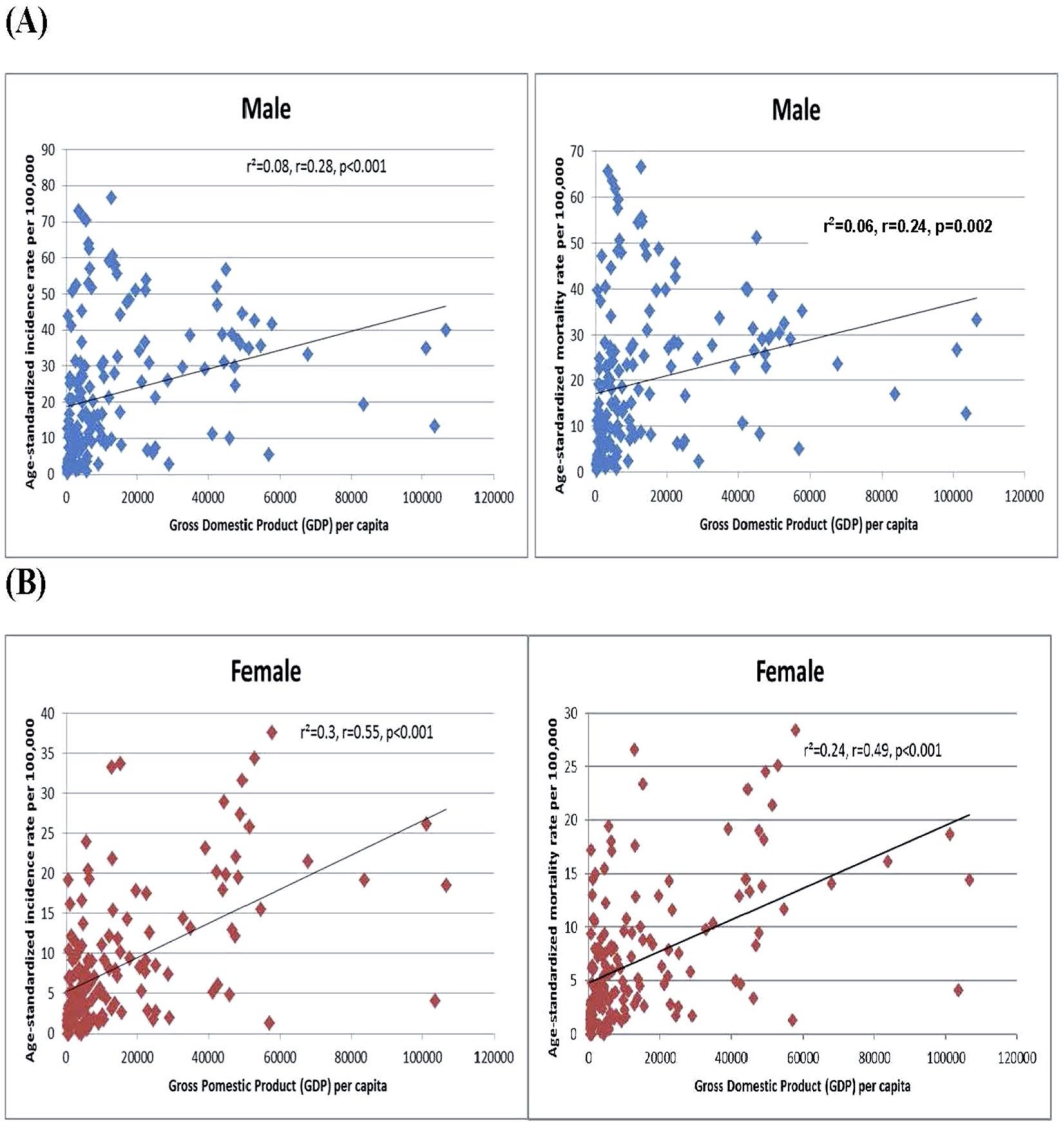
(**A**) Correlation between age-standardized lung cancer incidence (left) and mortality (right) and Gross Domestic Product (GDP) per capita (Male). (**B**) Correlation between age-standardized lung cancer incidence (left) and mortality (right) and Gross Domestic Product (GDP) per capita (Female).

The age-standardized incidence rates of lung cancer increased with higher levels of HDI in men (r^2^ = 0.49, r = 0.70, p < 0.001) and women (r^2^ = 0.48, r = 0.70, p < 0.001) (Fig. 1A and B), and to a lesser extent these positive correlations were observed for GDP per capita (r^2^ = 0.08, r = 0.28, p < 0.001 and r^2^ = 0.30, r = 0.55, p < 0.001 for men and women, respectively) (Fig. 2A and B). The age-standardized mortality rates were statistically significantly correlated with HDI (r² = 0.45, r = 0.67, p < 0.001 [men]; r² = 0.45, r = 0.67, p < 0.001 [women]) and GDP per capita (r² = 0.06, r = 0.24, p = 0.002 [men]; r² = 0.24, r = 0.49, p < 0.01 [women]).

### Temporal trends in incidence of lung cancer

**Figure 3:**
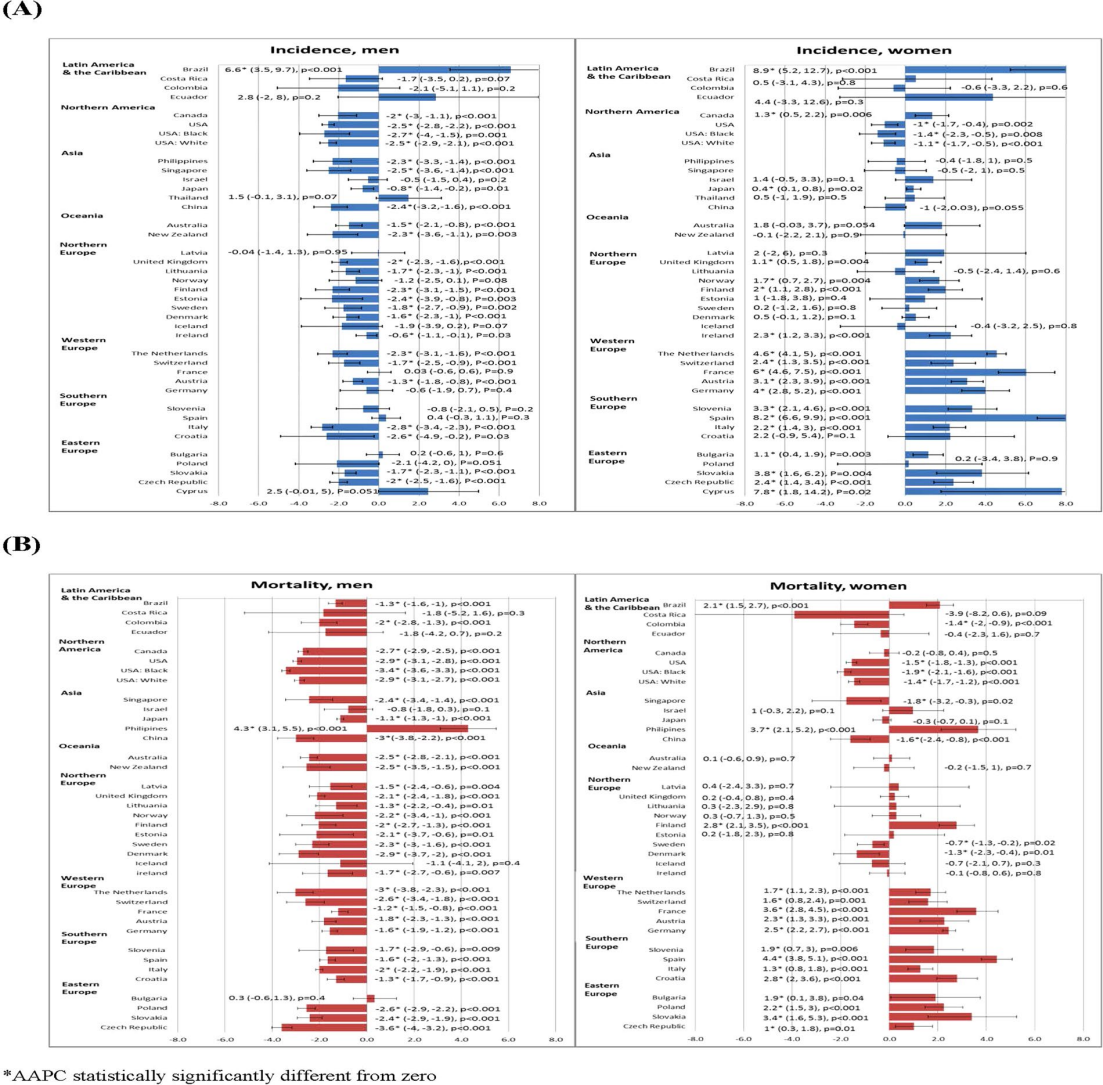
(**A**) The Average Annual Percent Change (AAPC) in the incidence of lung cancer in men (left) and women (right) in the most recent 10 years (the numbers in parentheses represent the 95% confidence intervals of the Average Annual Percentage Change). (**B**) The Average Annual Percent Change (AAPC) in the mortality of lung cancer in men (left) and women (right) in the most recent 10 years (the numbers in parentheses represent the 95% confidence intervals of the Average Annual Percentage Change).

The temporal trends of incidence and mortality of lung cancer in 38 countries/regions were presented by continents and gender. Among men, there was only one country with increasing incidence, 22 countries with decreasing incidence, and 15 countries with stable incidence. Among women, there were 19 countries with increasing incidence, one with decreasing incidence, and 18 countries with stable incidence (Fig. 3A). Brazil (AAPC = 6.6, 95% C.I. 3.5, 9.7, p < 0.001) was the only country that showed an increasing trend in men, whilst the United States (AAPC = −1.0, 95% C.I. −1.7, −0.4, p = 0.002) was the only country that reported a declining trend in women. For countries with reductions in incidence rates among men, the AAPCs ranged from −2.8 (Italy) to −0.6 (Ireland). Among all countries that encountered increase in incidence among women, the AAPCs ranged from 0.4 (Japan) to 8.9 (Brazil). Spain (AAPC = 8.2, 95% C.I. 6.6, 9.9, p < 0.001) and Cyprus (AAPC = 7.8, 95% C.I. 1.8, 14.2, p = 0.02) showed substantial increase in incidence among women.

### Temporal trends in lung cancer mortality

For mortality, there was one country with increasing trends, 30 countries with decreasing trends, and 5 countries with stable trends in men. There were 16 countries with increasing mortality trends, 6 countries with decreasing trends and 14 countries with stable trends in women (Fig. 3B). The Philippines was the only country with mortality increase in men (AAPC = 4.3, 95% C.I. 3.1, 5.5, p < 0.001) and women (AAPC = 3.7, 95% C.I. 2.1, 5.2, p < 0.001). Brazil (AAPC = 2.1, 95% C.I. 1.5, 2.7, p < 0.001), Finland (AAPC = 2.8, 95% C.I. 2.1, 3.5, p < 0.001), and all countries in Western Europe, Southern Europe and Eastern Europe were found to have increasing mortality in women. The AAPCs of mortality decline ranged from −1.1 (Japan) to −3.6 (Czech Republic) in men, and that of mortality increase ranged from 1 (Czech Republic) to 4.4 (Spain) in women.

## Discussion

This study presents a comprehensive epidemiological analysis of the global profiles of lung cancer incidence and mortality based on high quality data. Geographical variation in incidence and mortality was substantial between continents. The ASR of incidence and mortality were both positively correlated with levels of human development, and to a lower degree, country-specific GDP per capita. The correlation coefficients for HDI for the incidence/mortality of lung cancer were high. Most countries included in this study showed declining incidence in men (22 out of 38) and increasing incidence (19 out of 38) in women. Similarly for mortality, a high proportion of countries reported a decreasing trend in men (30 out of 36) and increasing trend in women (16 out of 36). These findings suggested gender and socioeconomic disparities of lung cancer incidence and mortality.

It was found that countries with higher levels of HDI reported higher incidence and mortality of lung cancer. HDI is a composite index of life expectancy at birth, access to knowledge (a combination of adult literacy rate and enrolment rates of primary to tertiary education), and income per capita adjusted for purchasing-power parity^19^. Bray and colleagues performed a study evaluating the changing patterns of cancer according to different levels of HDI based on the GLOBOCAN 2008 database^30^. It was found that cancers of the lungs, female breast, prostate and colorectum accounted for over half of the overall cancer burden in the highest HDI regions, and the lifetime cumulative risk of these four cancers were all more than 3% in higher HDI areas. All four cancers are not associated with infectious causes. The higher incidence and mortality of lung cancer in countries with higher HDI has been explained by the greater westernization effect where tobacco and air pollution due to industrialization were more common in these more developed nations. On the other hand, the systems of reporting epidemiological figures in these higher HDI countries are generally more robust, and it remains uncertain whether this observation could be attributed to reporting biases^5^. Hence, data should be interpreted with caution when these incidence and mortality figures were compared across countries.

Incidence and mortality rates of lung cancer have been declining in men and rising in women on a global scale, which corroborate with the findings from previous studies^4,8^. This could be explained by the uptake and subsequent decline in male smoking prevalence^14^, which was followed by later smoking uptake by women in many high HDI countries. Indoor exposure to fumes from cooking and heating using coal or combustible materials in unventilated stoves could also increase lung cancer risk in women populations who had low smoking prevalence^31^.

This study presented and analyzed the most up-to-date epidemiological data on lung cancer, and quantified the geographical variations as well as trends in its incidence and mortality using data of high validity, completeness and comparability. We also adopted figures on national mortality that fulfilled criteria attaining at least WHO-defined medium levels of coverage and completeness. The IARCs estimation methods have been further refined in more recent years to take into account the increasing availability and quality of the source data. Nevertheless, some limitations should be addressed. Firstly, failure or under-ascertainment of cancer diagnoses could lead to bias in cancer registration, especially in relatively less-developed nations^5^. Figures in regional cancer registries could be underestimated owing to limited local facilities. But in countries where estimates were based on a single cancer registry in more urbanized, resource privileged areas, the presented figures could be an overestimation if the countries consist of extensive rural populations. Also, only one-third and one-fifth of the world’s countries, respectively, reported incidence and mortality data of high quality. As a result, the incidence and mortality data are constrained with respect to geographical coverage, in particular the resource-deprived countries. Secondly, previous studies used age- and gender-standardized rates^32,33^, which can provide population-based cancer incidence and mortality rates. The study by Rauma *et al*.^32^ compared the long-term health-related quality of life between lung cancer survivors after surgery and the general public. They matched individuals recruited from the Finnish general population where the sample was weighted to reflect patients’ age and gender distribution. The study by Dawe and colleagues^33^ examined the incidence of non-small cell lung cancer and the incidence cases were also standardized by age and sex. This was not feasible in the present study as most databases do not have data on gender-standardized rates. One implication is on the increasing mortality rates among women but decreasing mortality rates among men in Western, Southern and Eastern European countries. Should age- and gender-standardized rates be used, the changes in AAPC of mortality could become marginal. The overall change in AAPC of age- and gender-standardized incidence rates might also be modest - as the increase in incidence rates among women in most countries could be offset by the incidence decrease in men. Thirdly, this is an ecological study, and the presence of ecological fallacy might lead to biases. We have deduced the correlation between incidence/mortality rates and HDI/GDP from the correlation of the variables collected for the group to which those individuals belong. Because data at individual level on socioeconomic status and the various potential confounders were not available, one should be cautious when the correlation between the cancer incidence/mortality rates and the socioeconomic indexes was interpreted. As discussed above, the correlation might be due to more robust reporting system in countries with higher HDI or GDP per capita. Finally, there might exist attribution bias due to difficulty in ascertainment of mortality cause, as in some cases where patients died of metastatic lung disease whilst the primary cancers were not identified in some cases.

In summary, the incidence and mortality rates of lung cancer were higher in countries with higher socioeconomic development, and most countries examined in this study showed a declining trend in men but increasing trend in women. With population growth, clinicians and policy-makers might expect a further substantial rise in its global health burden – particularly in women and high HDI countries. Hence, more healthcare resources will be needed to cope with the treatment and follow-up consultations of patients diagnosed with the cancer. These findings could also act as a reference point where preventive strategies like the WHO Framework Convention on Tobacco Control should be further reinforced in higher HDI countries, through taxation, smoke-free areas, monitoring, cessation assistance, education about harms of tobacco, and bans on tobacco advertising^34^. Many developing countries are suffering from an increasing trend especially among women, and tobacco control strategies are needed as these are typically the target for the tobacco industry’s search for a new customer base. These measures have been shown to be effective in smoking cessation in a number of world regions. Future studies should explore the underlying reasons for these epidemiological trends, which could offer further insights into the specific etiological factors of lung cancer. The correlations reported in this study should also be validated in future studies based on individual level data.

## Electronic supplementary material

Supplementary Figures
