# Supplementary material for: Incidence and mortality of lung cancer: global trends and association with socioeconomic status
Source: Sci Rep. 2017 Oct 30;7:14300. doi: 10.1038/s41598-017-14513-7 (PMC5662733; doi:10.1038/s41598-017-14513-7)
Supplement: Supplementary file 1 — Supplementary Figures [file 41598_2017_14513_MOESM1_ESM.pdf]

Incidence and mortality of lung cancer: global trends and association with socioeconomic status

Martin CS Wong MD, MPH [a, b], Xiang Qian Lao PhD [c], Kin-Fai Ho PhD [c], William B Goggins DSc (Harvard) [d], Shelly LA Tse PhD[c]

a. Division of Family Medicine and Primary Healthcare, School of Public Health and Primary Care, Faculty of Medicine, Chinese University of Hong Kong

b. Family Medicine and Primary Health Care, Hospital Authority, Hong Kong

c. Division of Occupational and Environmental Health, School of Public Health and Primary Care, Faculty of Medicine, Chinese University of Hong Kong

d. Division of Biostatistics, School of Public Health and Primary Care, Faculty of Medicine, The Chinese University of Hong Kong.

Corresponding Author: Martin C.S. Wong

BMedSc (Hons), MSc (Hons), MBChB, MD, MPH, MBA, FRSPH, FRSP, FRIPH, FRACGP, FHKCFP, DCH, FHKAM

Professor of Family Medicine and Primary Healthcare, JC School of Public Health and Primary Care, Faculty of Medicine, The Chinese University of Hong Kong

Consultant (Honorary), Department of Family Medicine, Hospital Authority

Tel: (852) 2252 8782 **Fax**: (852) 2606 3500; **Email**: wong_martin@cuhk.edu.hk

Address: 4/F, School of Public Health, Prince of Wales Hospital, Shatin, N.T., Hong Kong

7/F, Lui Che Woo Clinical Science Building, Prince of Wales Hospital, Shatin, NT, HKSAR.

Supplementary Figure 1 **The age-standardized incidence and mortality rates (World) of lung cancer in 2012**

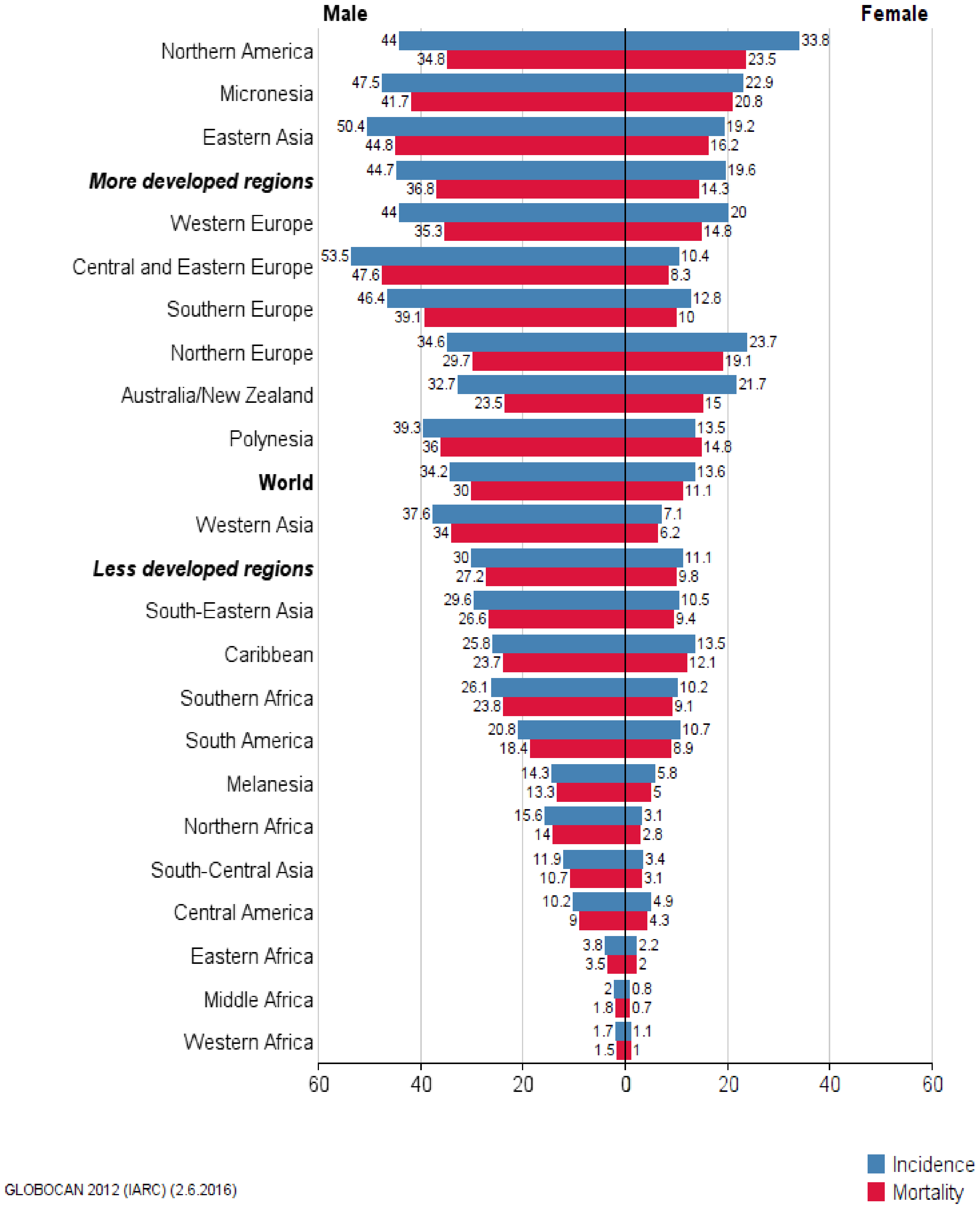

Source: Ferlay J, Soerjomataram I, Ervik M et al. GLOBOCAN 2012 v1.0, Cancer Incidence and Mortality Worldwide. IARC Cancer Base No. 11. Lyon, France: International Agency for Research on Cancer, 2013.

Supplementary Figure 2 Findings from the joinpoint regression analysis of the global incidence rates of lung cancer (Left: Male, Right: Female)

1) Latin America and the Caribbean

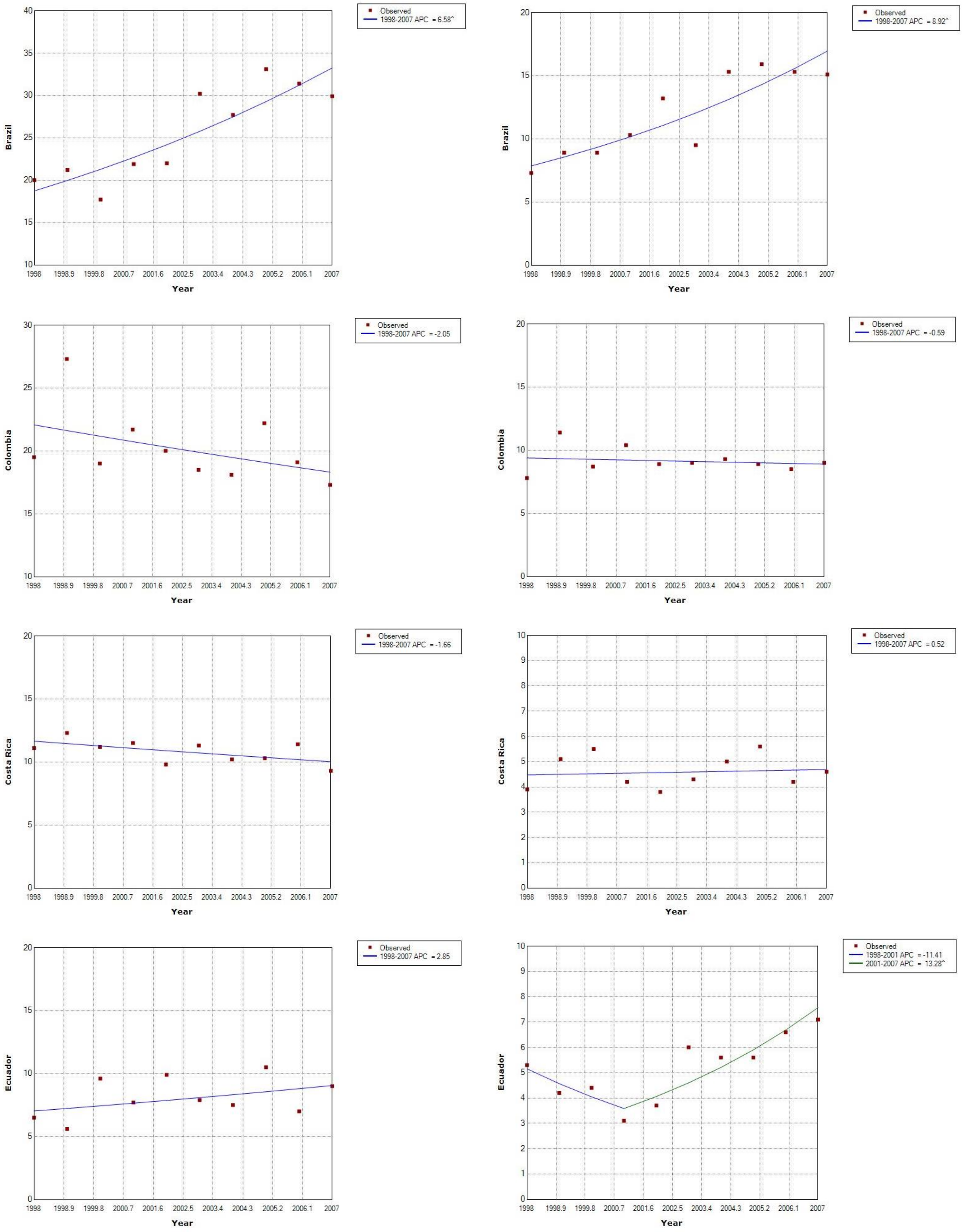

2) Northern America

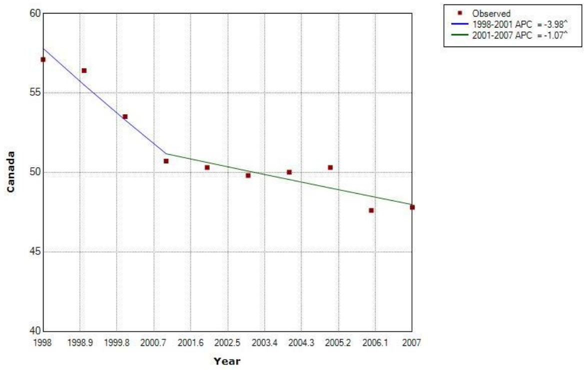

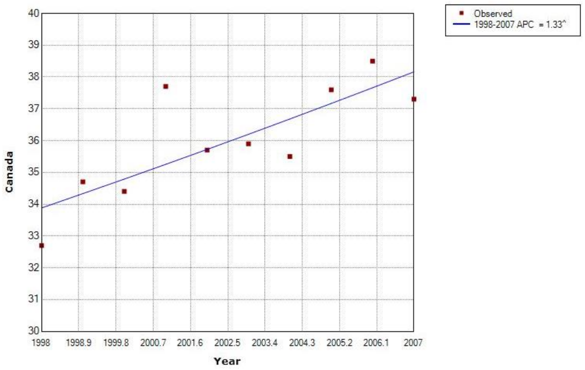

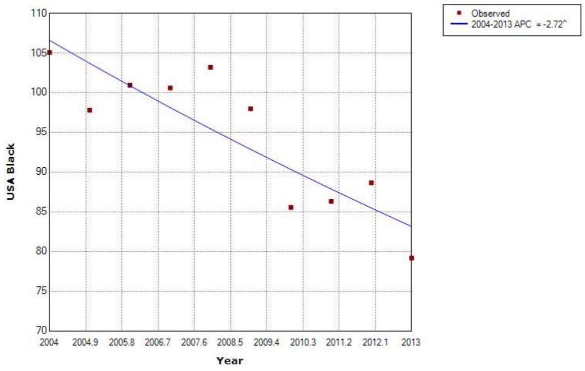

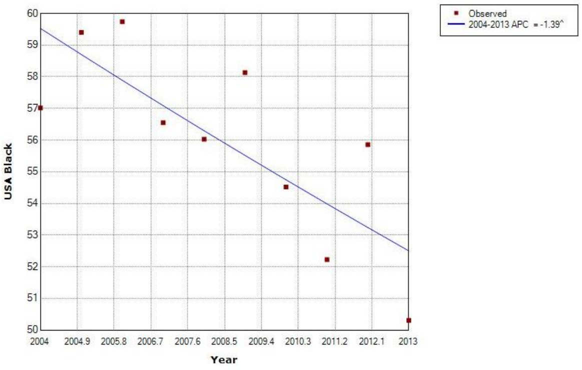

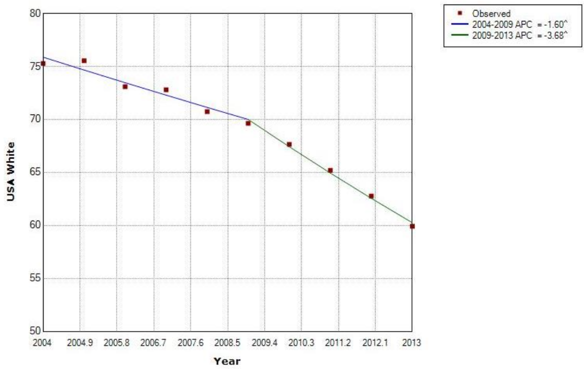

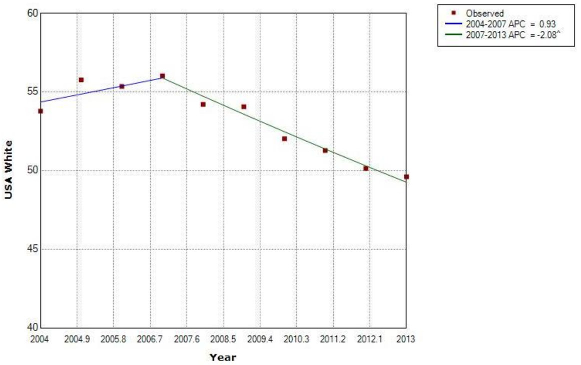

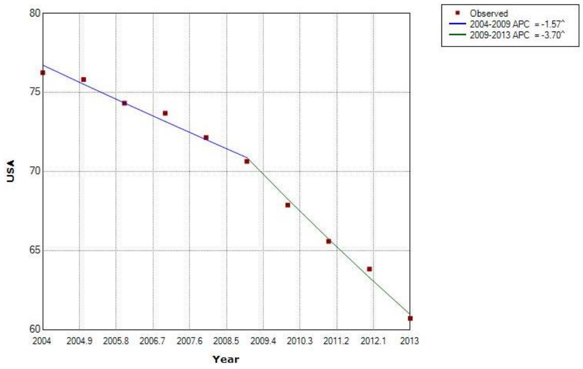

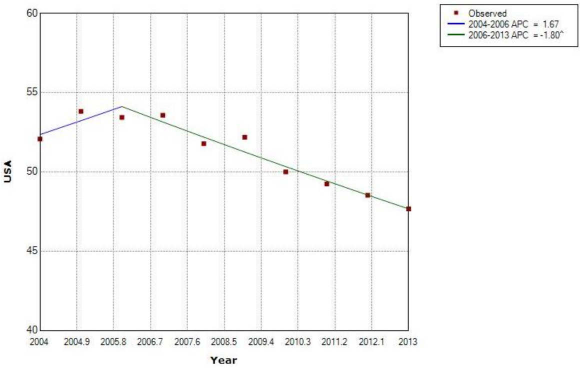

3) Asia

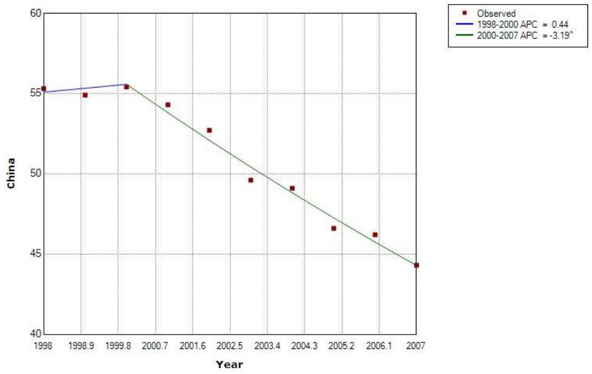
Observed
1998-2000 APC = 0.44
2000-2007 APC = -3.19^
China
Year

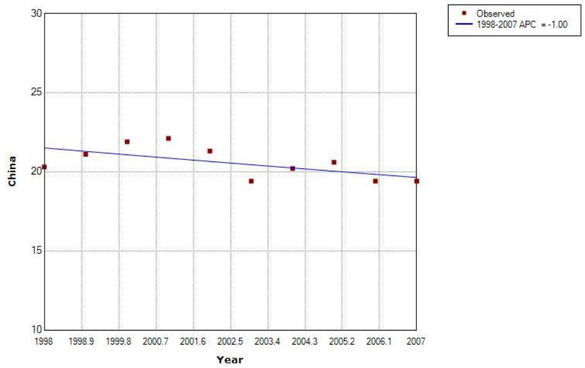
Observed
1998-2007 APC = -1.00
China
Year

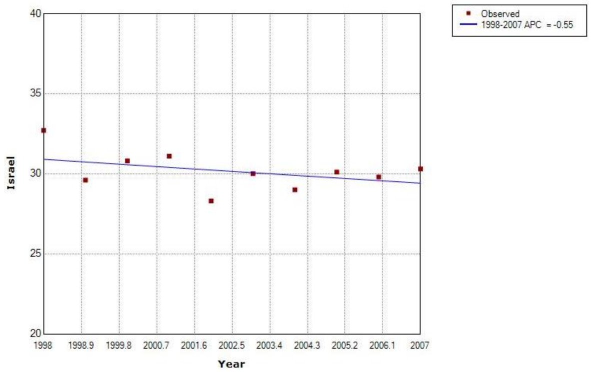
Observed
1998-2007 APC = -0.55
Israel
Year

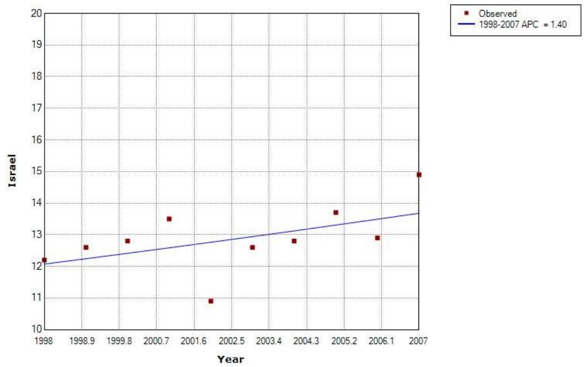
Observed
1998-2007 APC = 1.40
Israel
Year

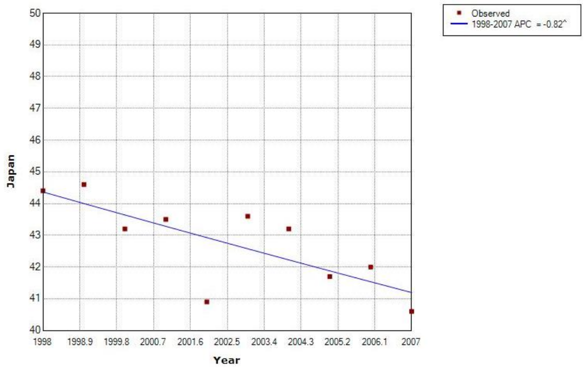
Observed
1998-2007 APC = -0.82^
Japan
Year

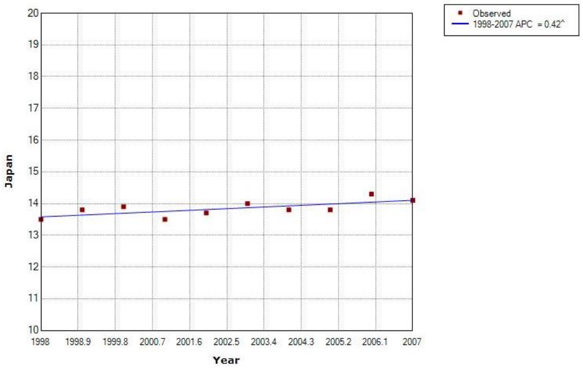
Observed
1998-2007 APC = 0.42^
Japan
Year

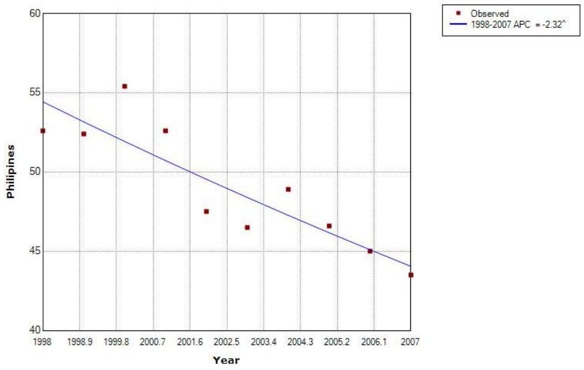
Observed
1998-2007 APC = -2.32^
Philipines
Year

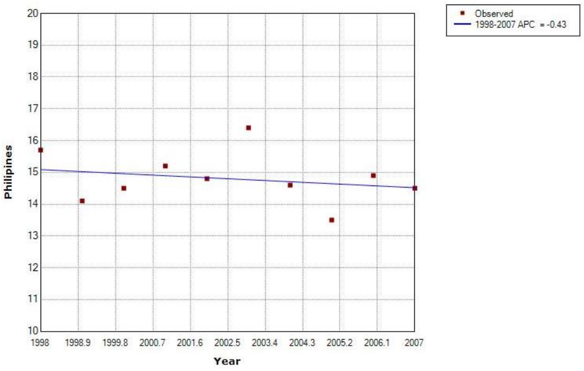
Observed
1998-2007 APC = -0.43
Philipines
Year

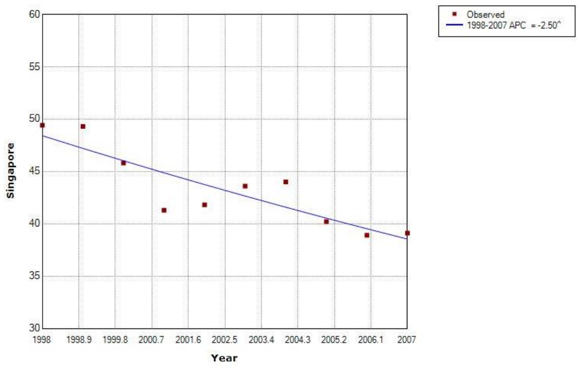
Observed
1998-2007 APC = -2.50^
Singapore
Year

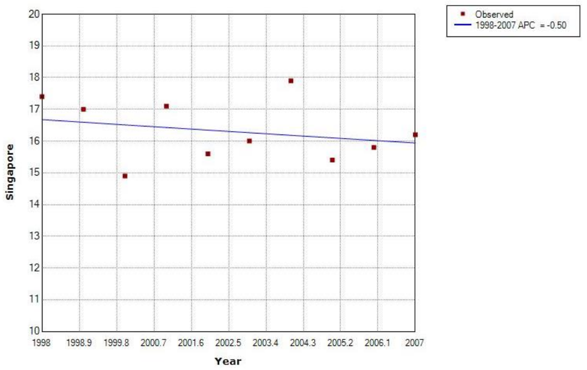
Observed
1998-2007 APC = -0.50
Singapore
Year

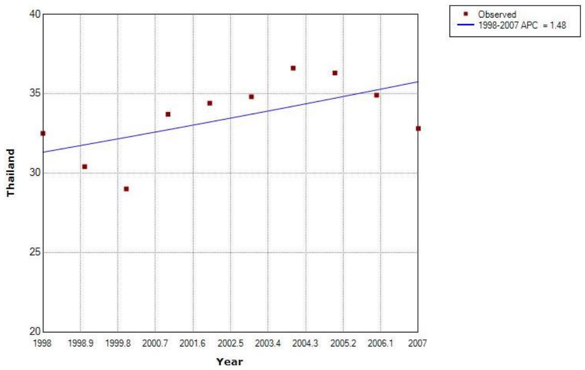
Observed
1998-2007 APC = 1.48
Thailand
Year

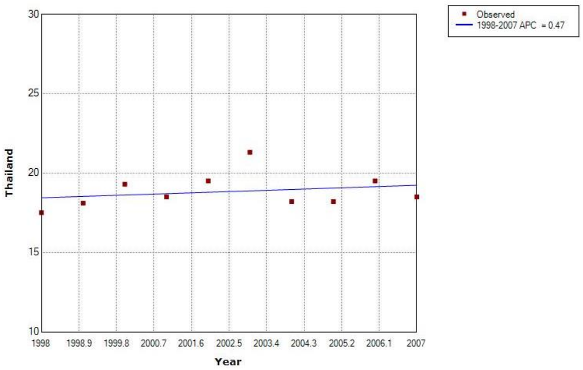
Observed
1998-2007 APC = 0.47
Thailand
Year

4) Oceania

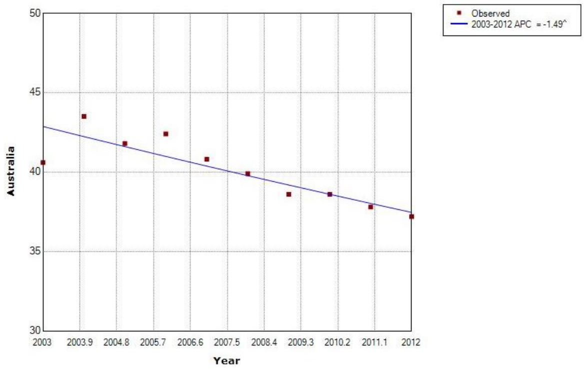
Observed
2003-2012 APC = -1.49^
Australia
Year

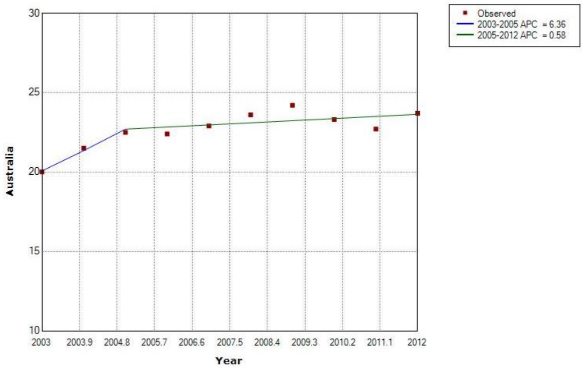
Observed
2003-2005 APC = 6.36
2005-2012 APC = 0.58
Australia
Year

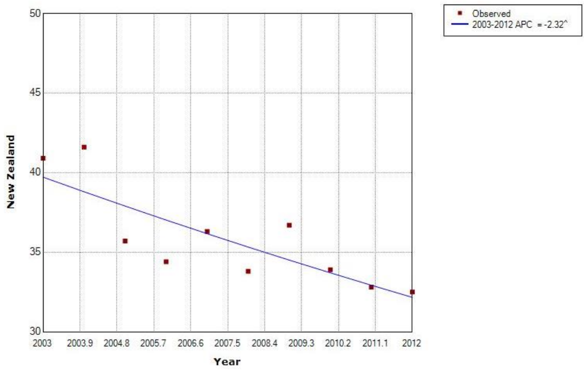
Observed
2003-2012 APC = -2.32^
New Zealand
Year

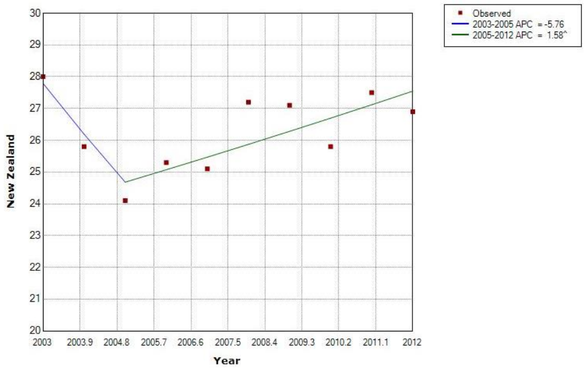
Observed
2003-2005 APC = -5.76
2005-2012 APC = 1.58^
New Zealand
Year

5) Northern Europe

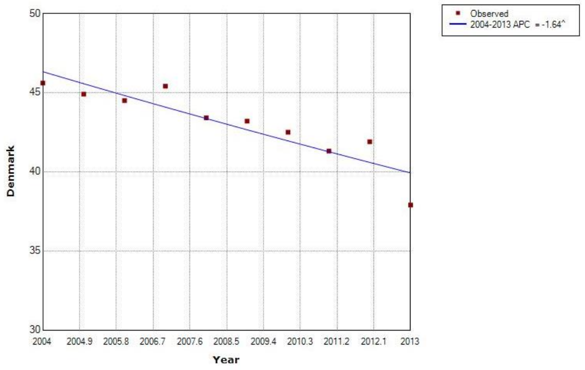

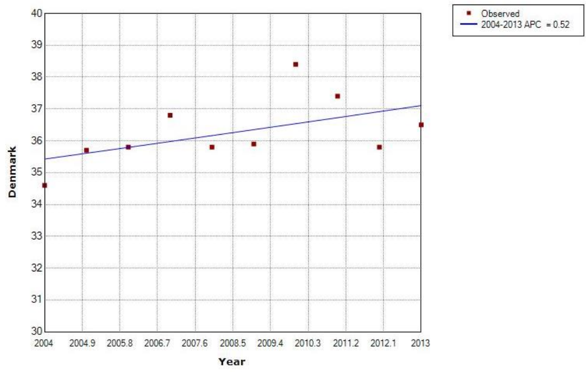

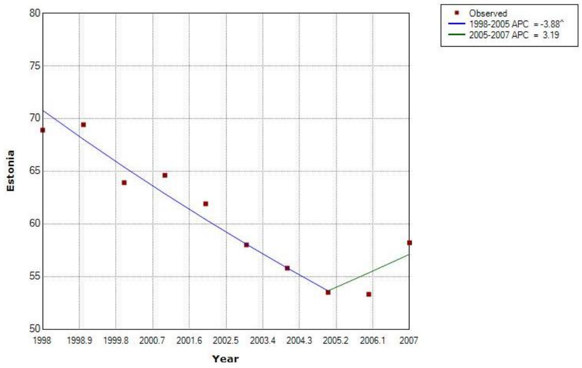

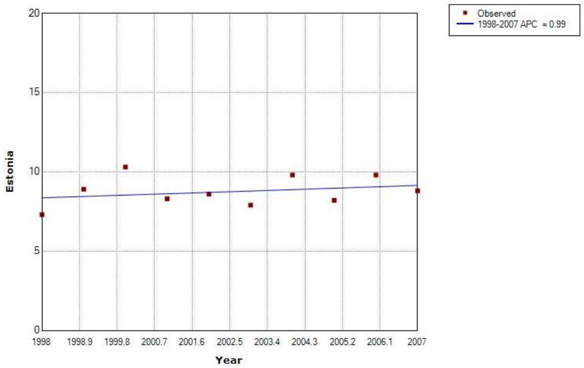

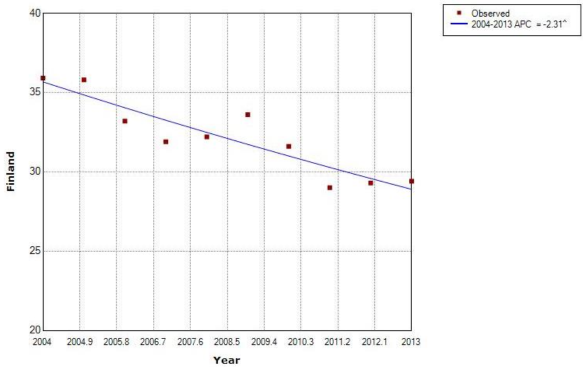

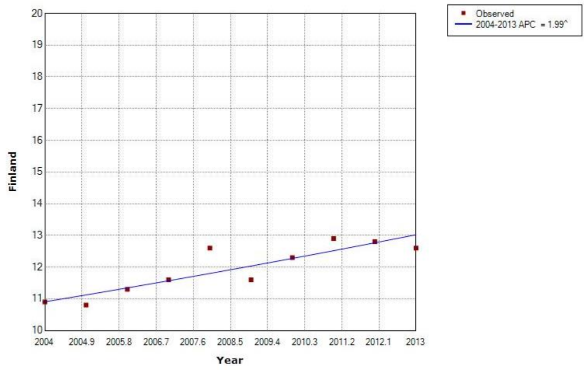

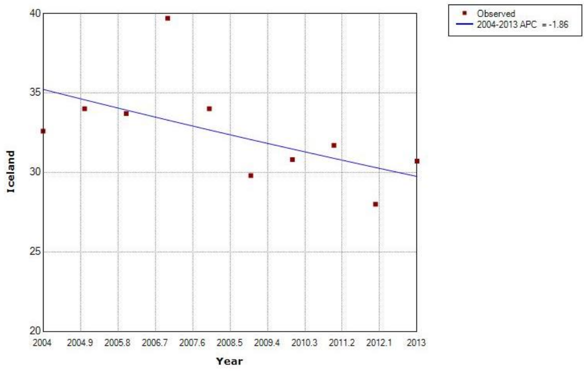

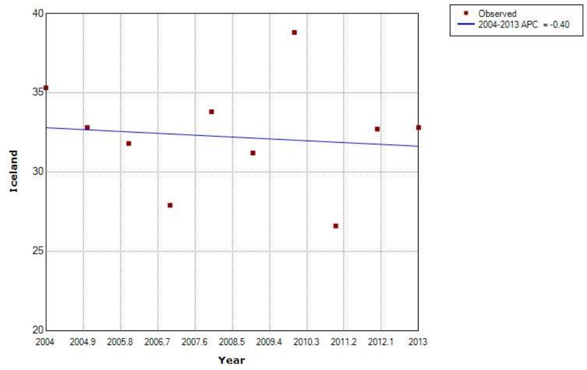

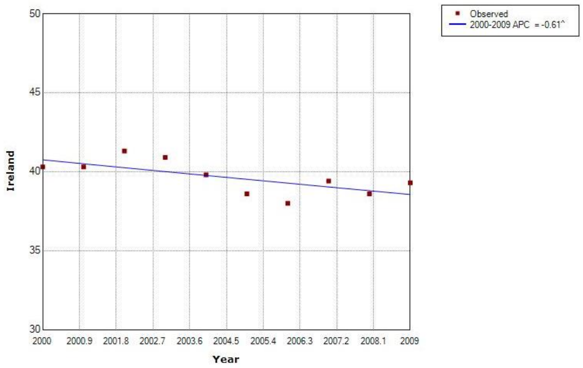
Observed
2000-2009 APC = -0.61^
Ireland
Year

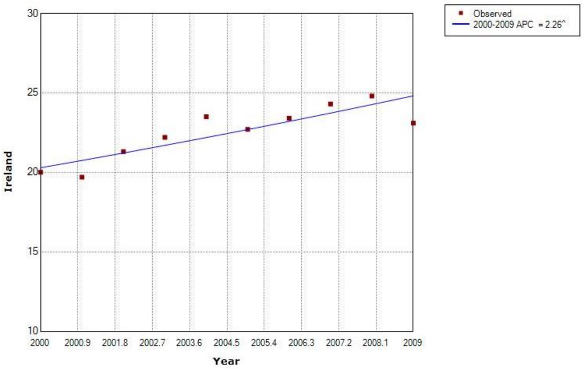
Observed
2000-2009 APC = 2.26^
Ireland
Year

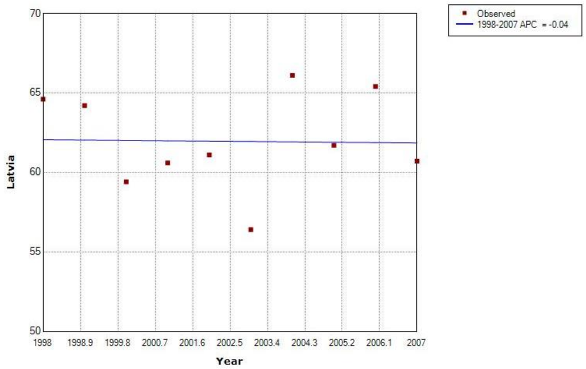
Observed
1998-2007 APC = -0.04
Latvia
Year

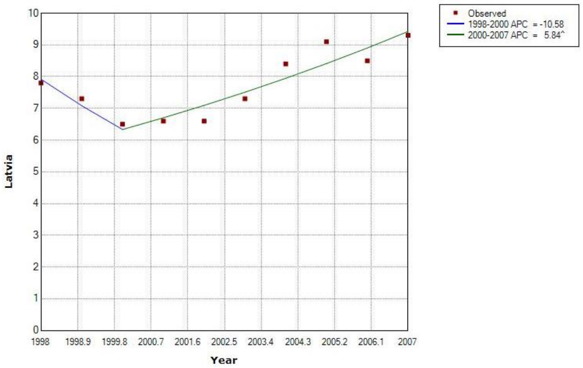
Observed
1998-2000 APC = -10.58
2000-2007 APC = 5.84^
Latvia
Year

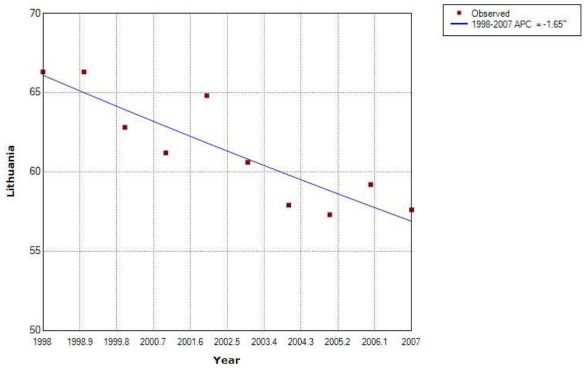
Observed
1998-2007 APC = -1.65^
Lithuania
Year

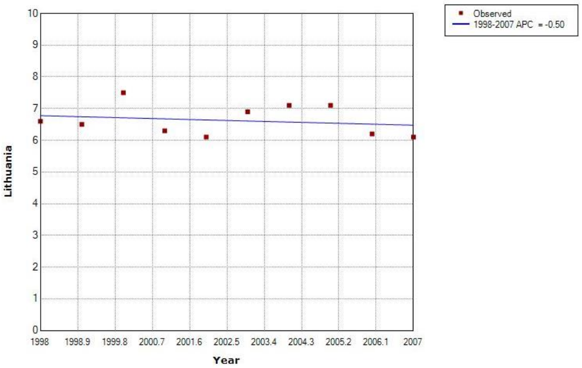
Observed
1998-2007 APC = -0.50
Lithuania
Year

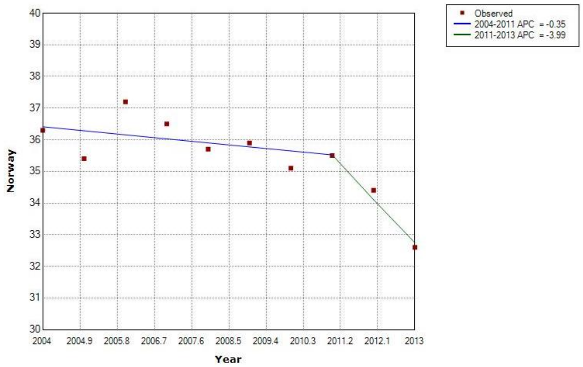
Observed
2004-2011 APC = -0.35
2011-2013 APC = -3.99
Norway
Year

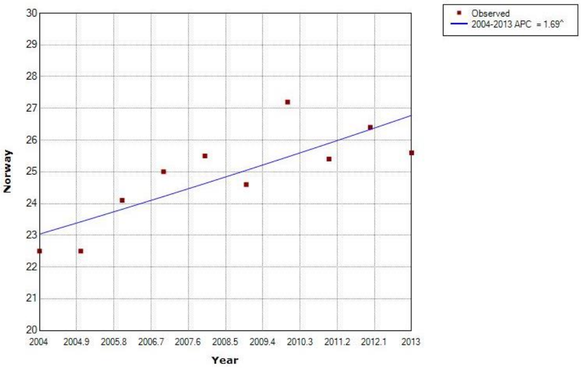
Observed
2004-2013 APC = 1.69^
Norway
Year

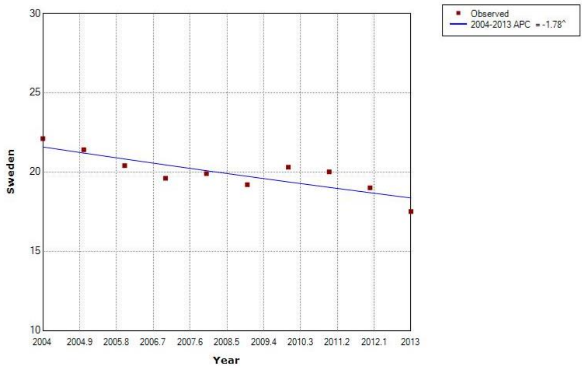
Observed
2004-2013 APC = -1.78^
Sweden
Year

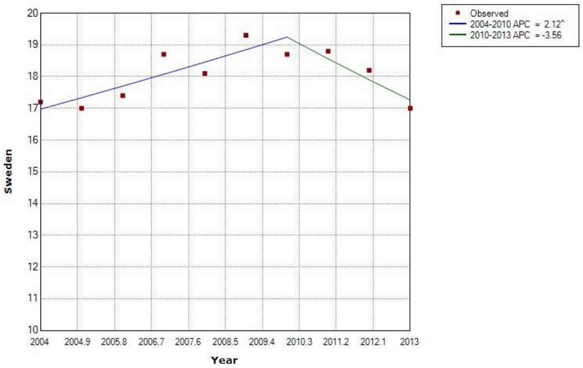
Observed
2004-2010 APC = 2.12^
2010-2013 APC = -3.56
Sweden
Year

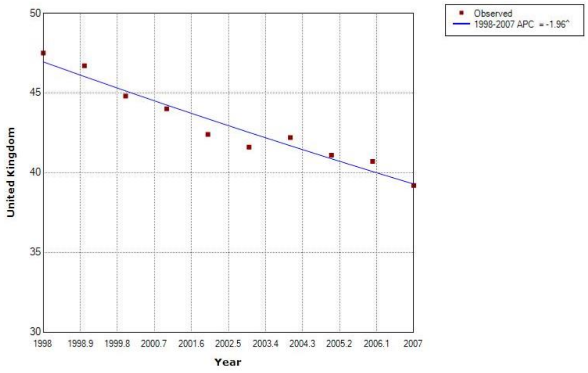

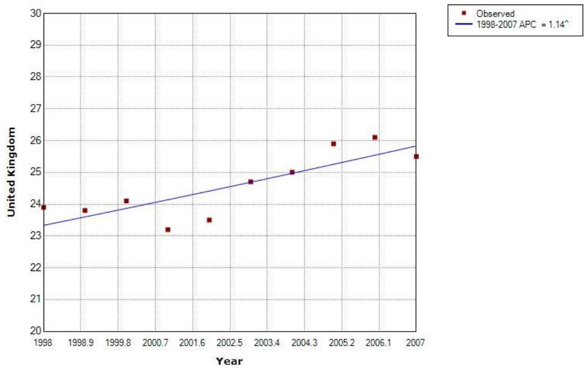

6) Western Europe

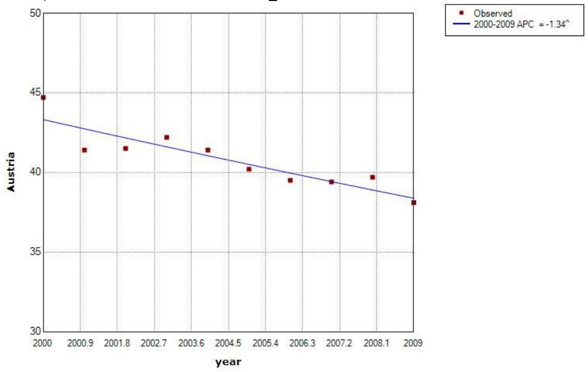

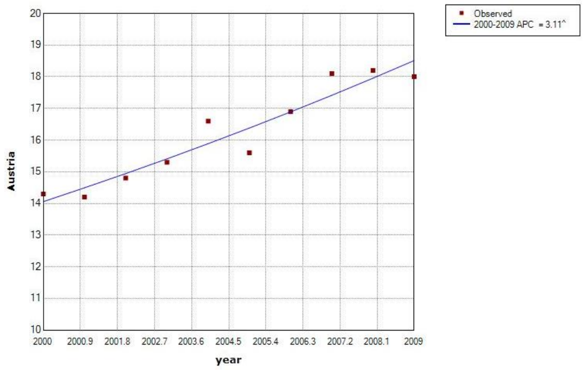

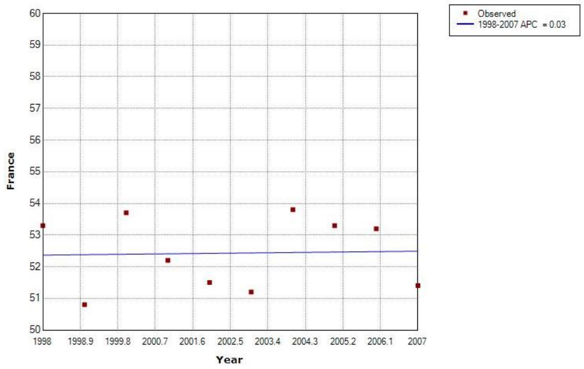

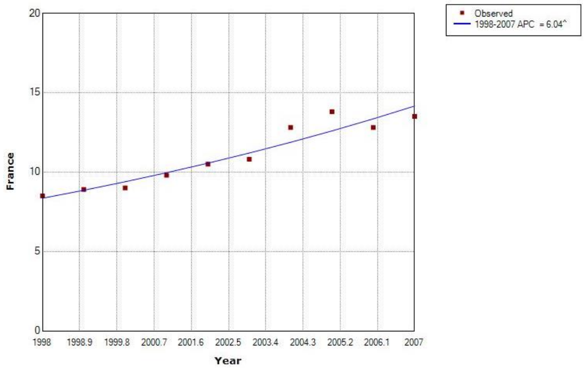

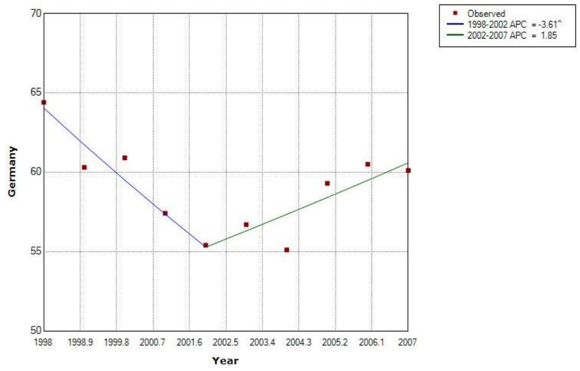

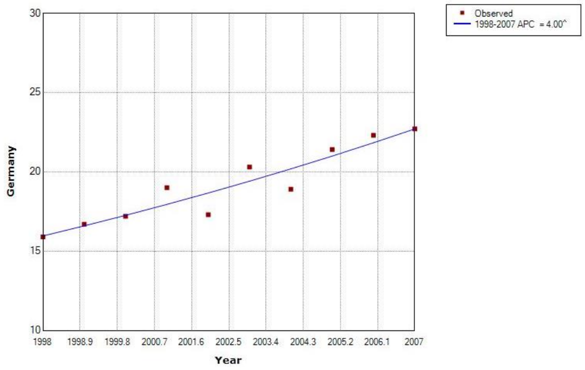

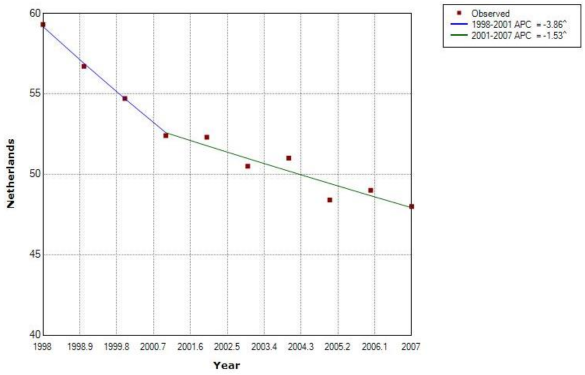

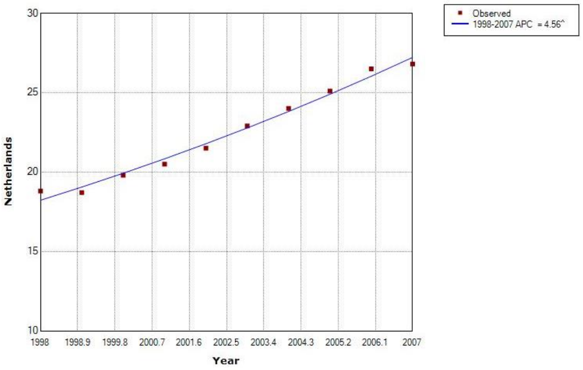

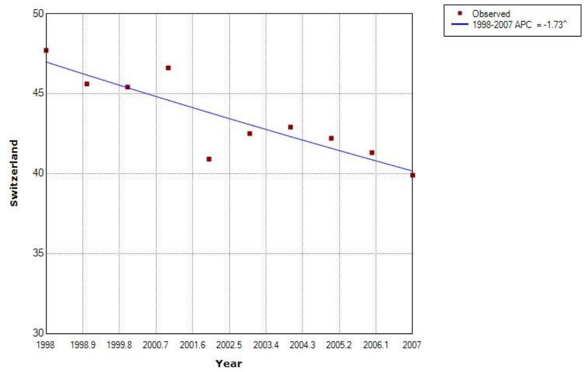

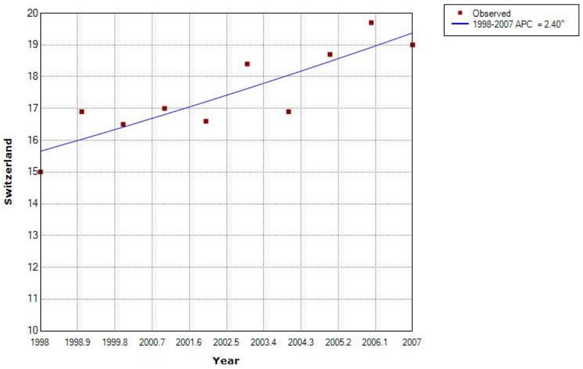

7) Southern Europe

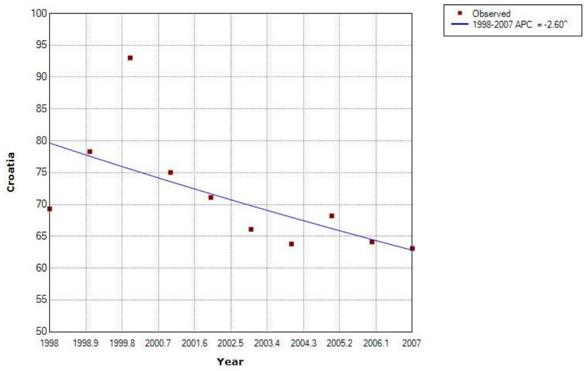

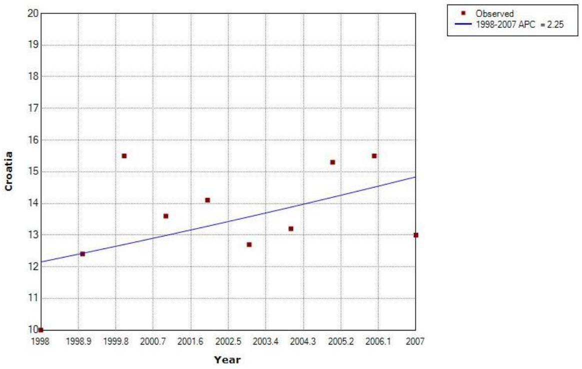

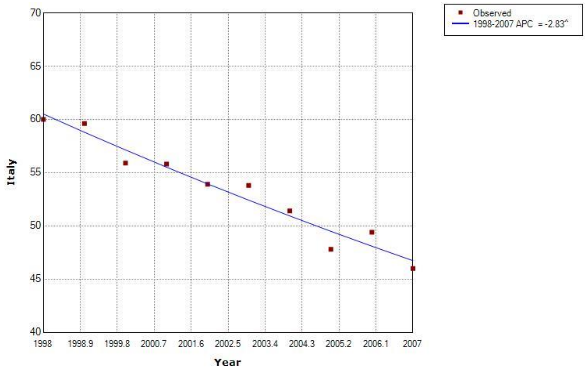

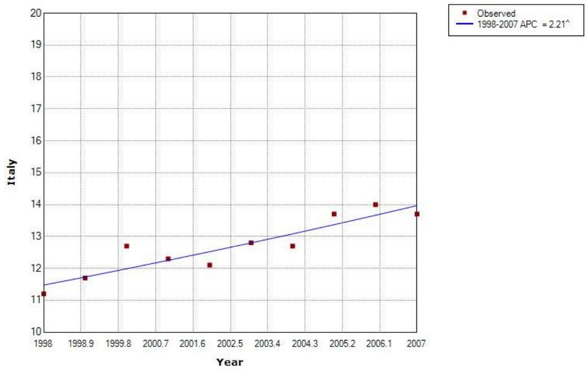

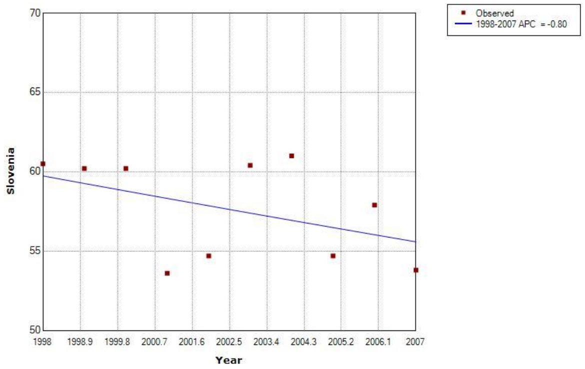

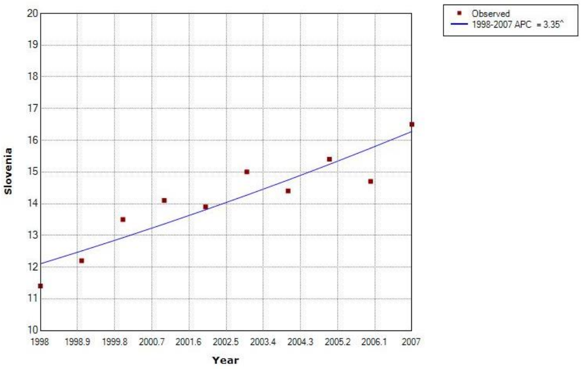

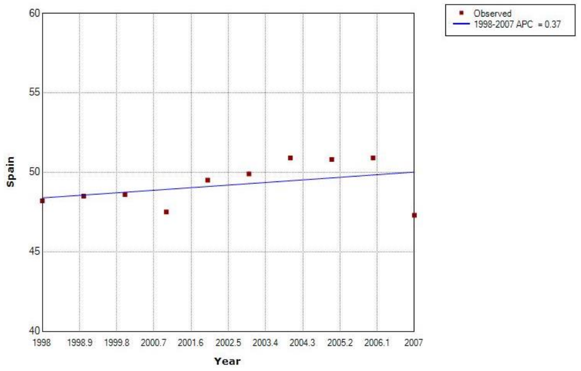

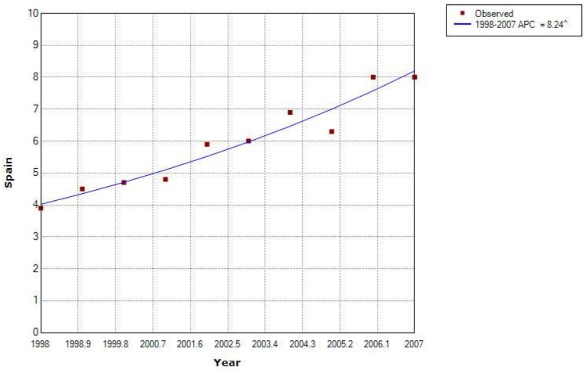

8) Eastern Europe

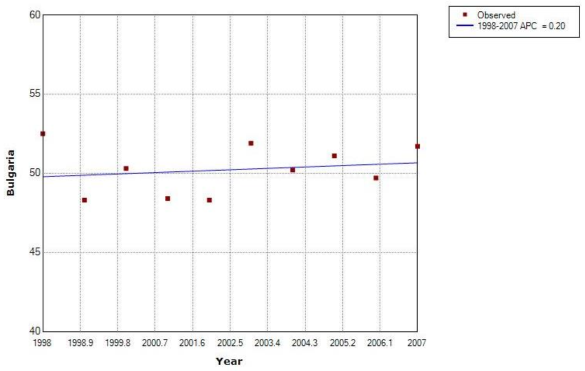

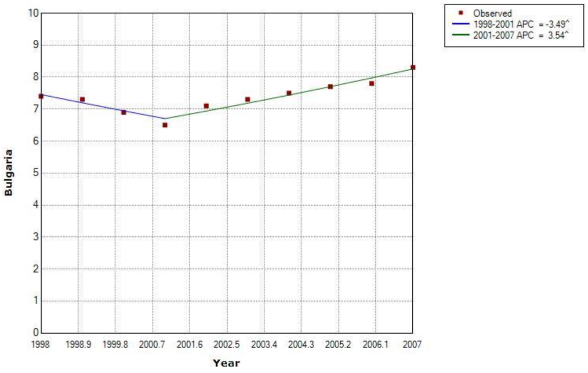

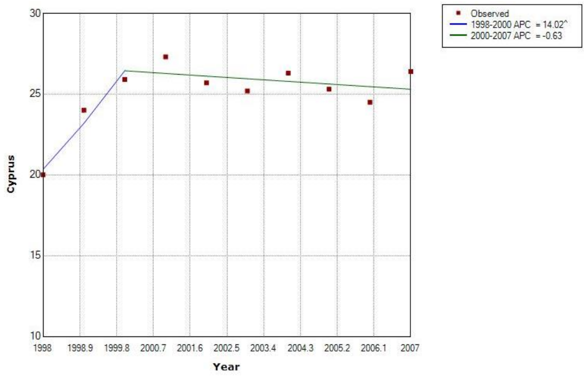

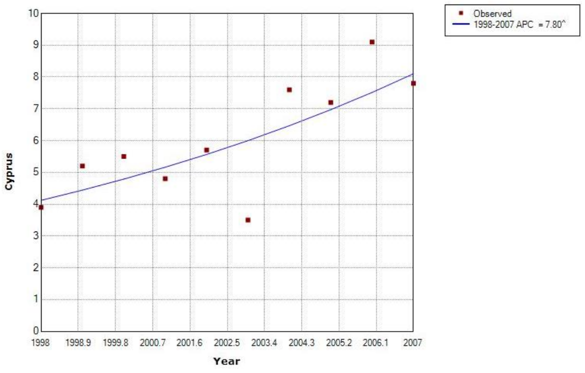

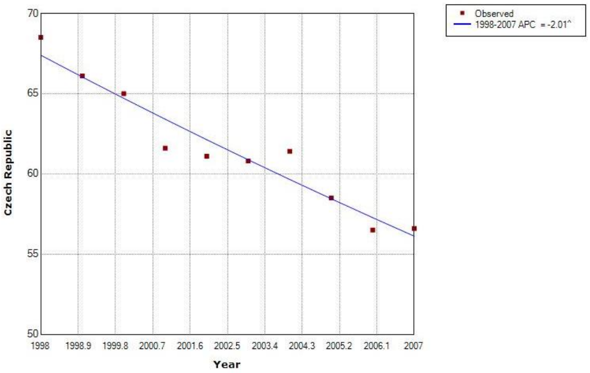
Observed
1998-2007 APC = -2.01^
Czech Republic
70
65
60
55
50
1998 1998.9 1999.8 2000.7 2001.6 2002.5 2003.4 2004.3 2005.2 2006.1 2007
Year

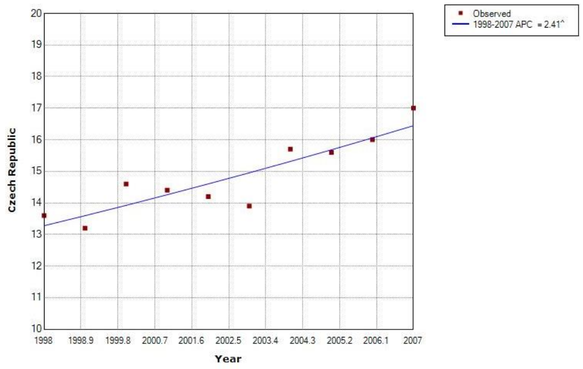
Observed
1998-2007 APC = 2.41^
Czech Republic
20
19
18
17
16
15
14
13
12
11
10
1998 1998.9 1999.8 2000.7 2001.6 2002.5 2003.4 2004.3 2005.2 2006.1 2007
Year

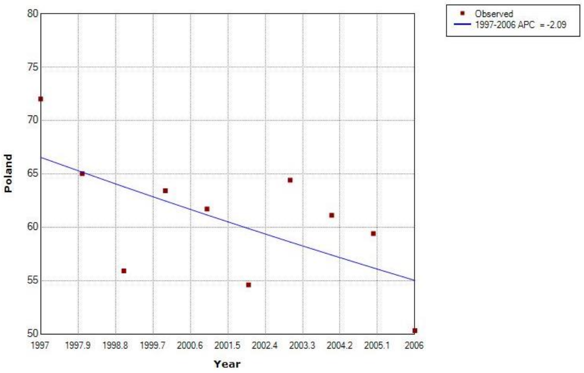
Observed
1997-2006 APC = -2.09
Poland
80
75
70
65
60
55
50
1997 1997.9 1998.8 1999.7 2000.6 2001.5 2002.4 2003.3 2004.2 2005.1 2006
Year

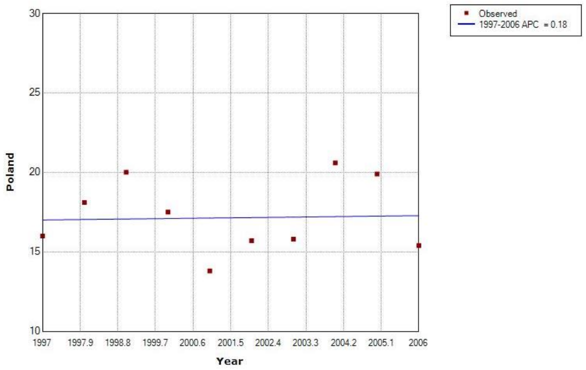
Observed
1997-2006 APC = 0.18
Poland
30
25
20
15
10
1997 1997.9 1998.8 1999.7 2000.6 2001.5 2002.4 2003.3 2004.2 2005.1 2006
Year

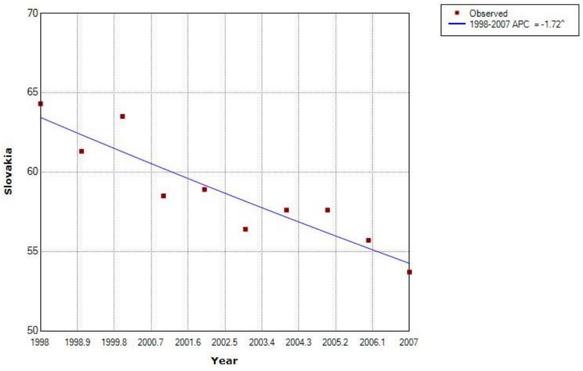
Observed
1998-2007 APC = -1.72^
Slovakia
70
65
60
55
50
1998 1998.9 1999.8 2000.7 2001.6 2002.5 2003.4 2004.3 2005.2 2006.1 2007
Year

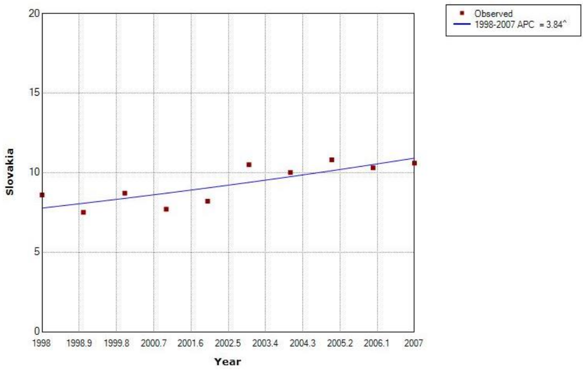
Observed
1998-2007 APC = 3.84^
Slovakia
20
15
10
5
0
1998 1998.9 1999.8 2000.7 2001.6 2002.5 2003.4 2004.3 2005.2 2006.1 2007
Year

Supplementary Figure 3 **Findings from the joinpoint regression analysis of the global mortality rates of lung cancer (Left: Male, Right: Female)**

1) **Latin America and the Caribbean**

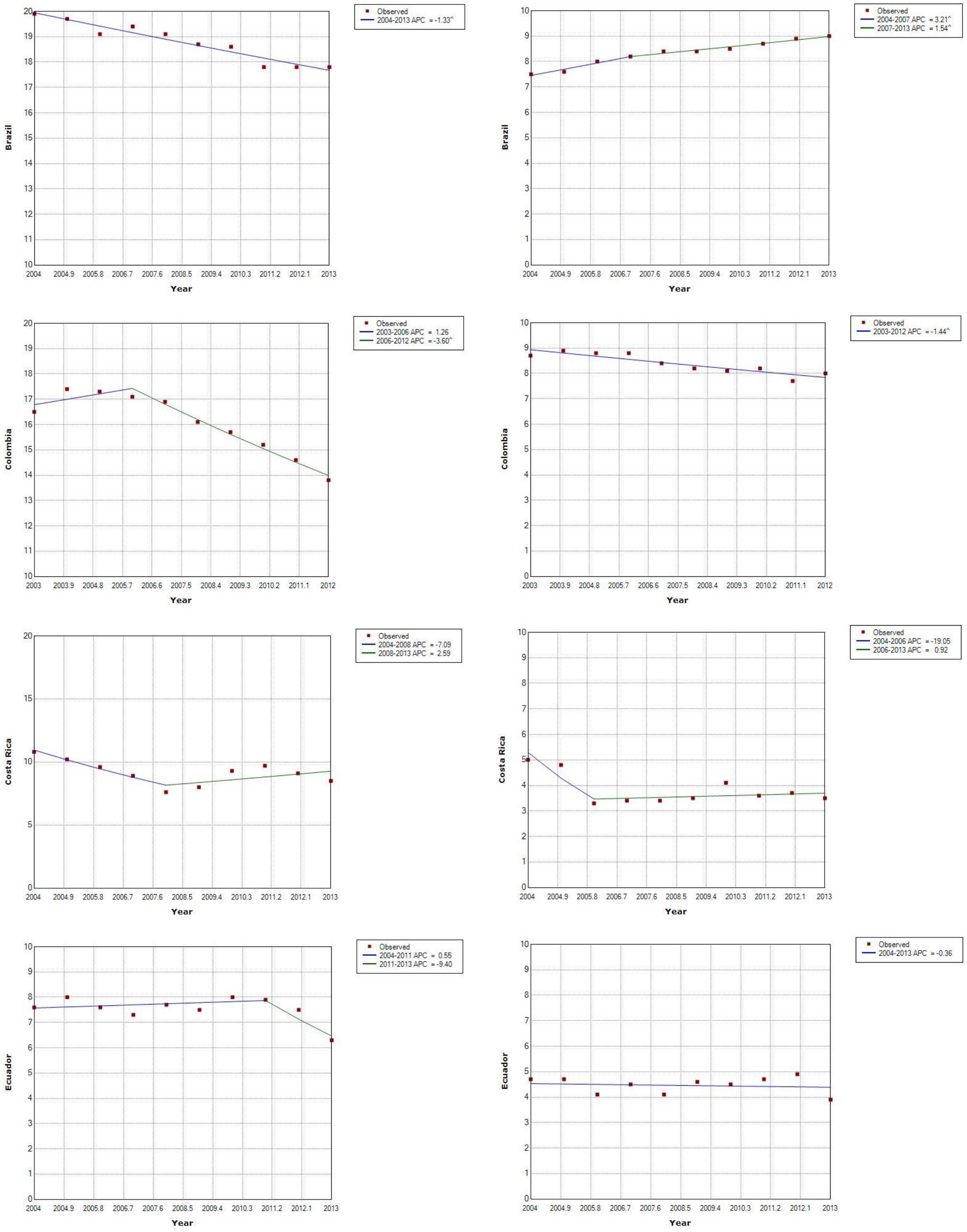

2) Northern America

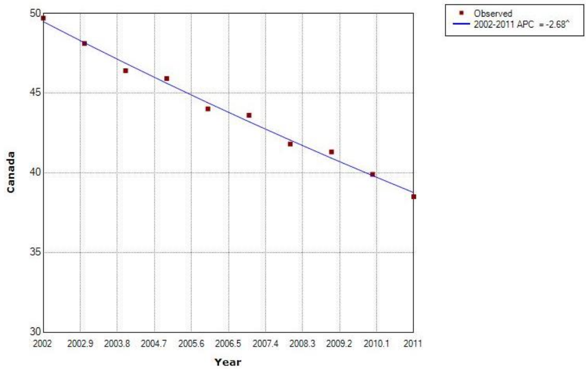

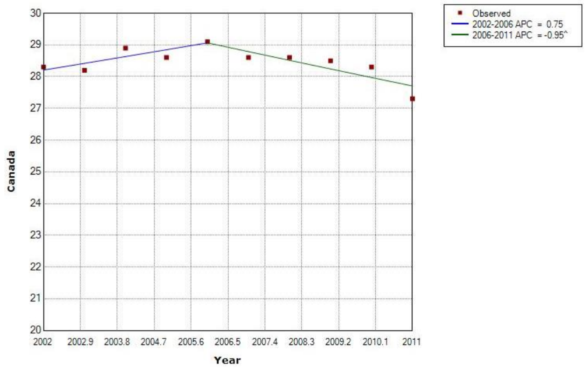

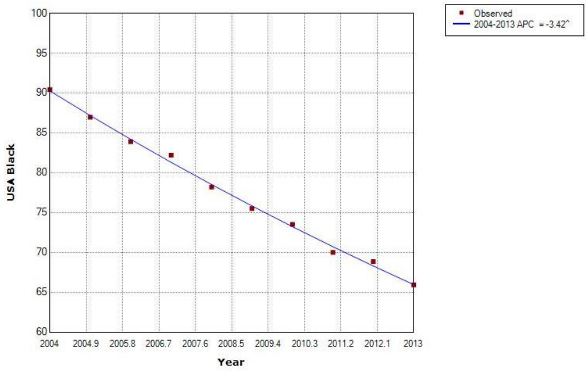

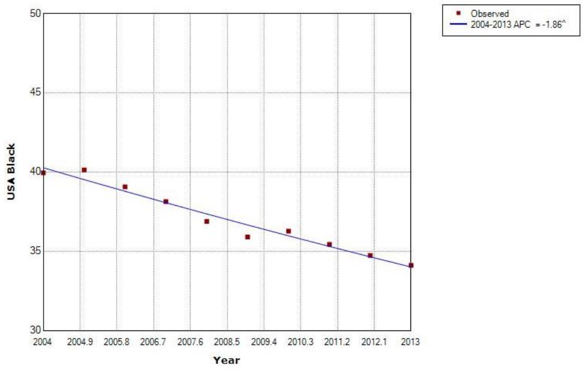

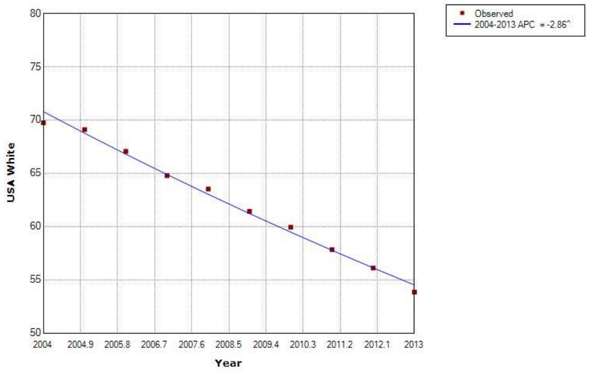

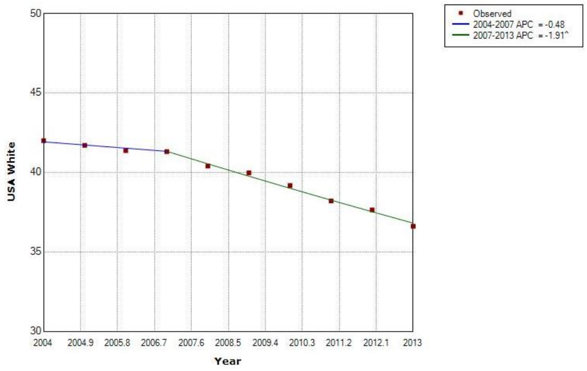

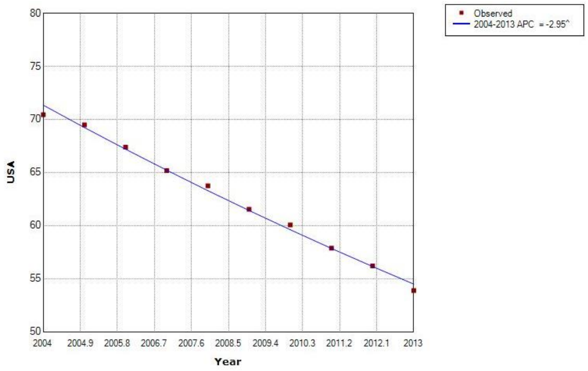

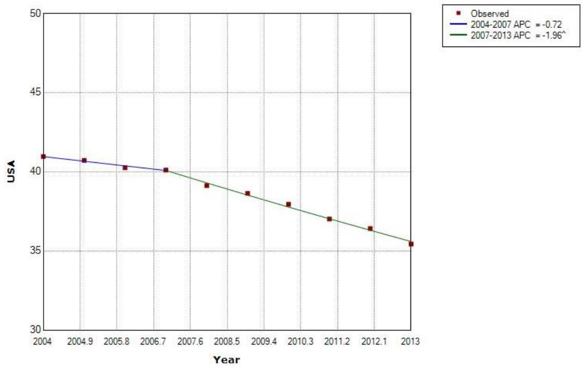

3) Asia

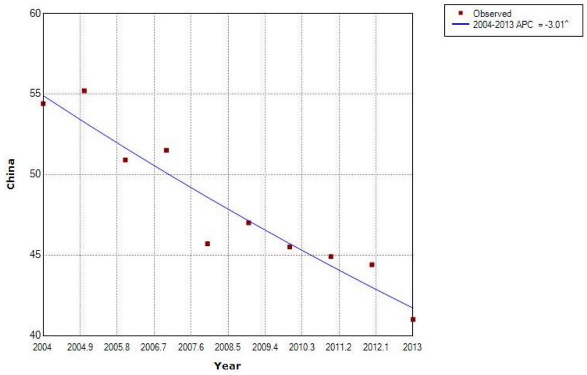
Observed
2004-2013 APC = -3.01^
China
Year

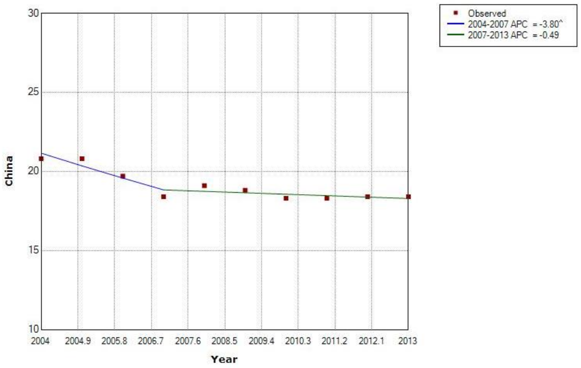
Observed
2004-2007 APC = -3.80^
2007-2013 APC = -0.49
China
Year

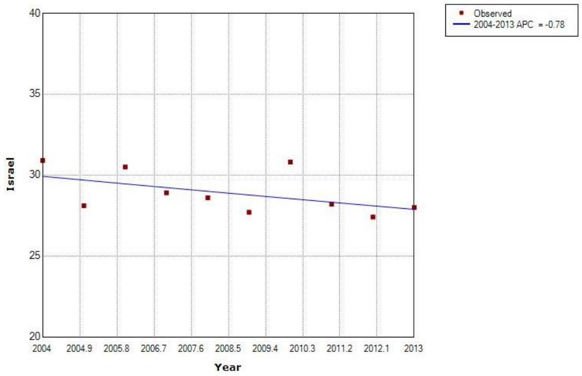
Observed
2004-2013 APC = -0.78
Israel
Year

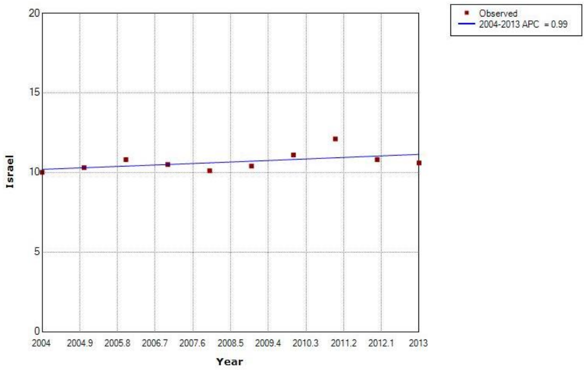
Observed
2004-2013 APC = 0.99
Israel
Year

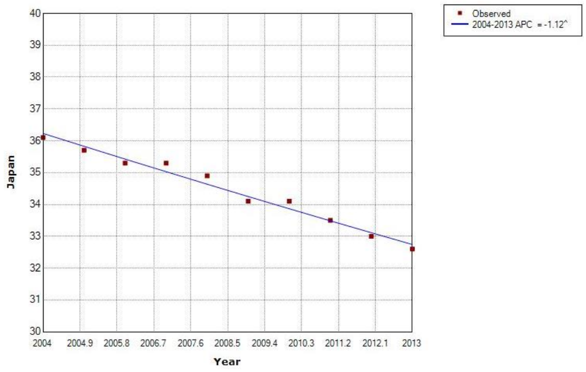
Observed
2004-2013 APC = -1.12^
Japan
Year

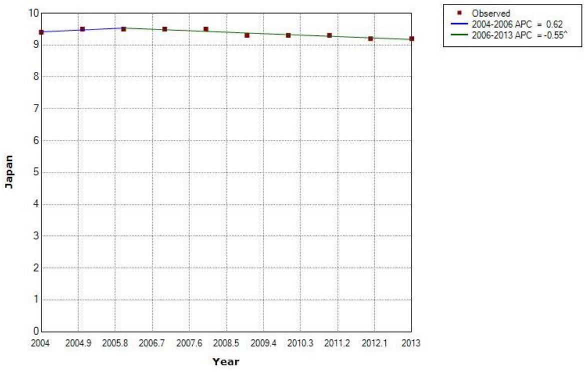
Observed
2004-2006 APC = 0.62
2006-2013 APC = -0.55^
Japan
Year

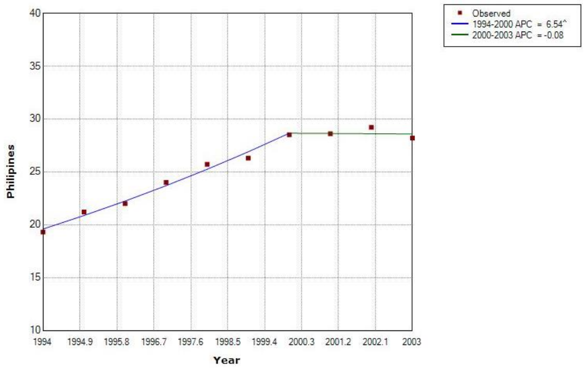
Observed
1994-2000 APC = 6.54^
2000-2003 APC = -0.08
Philipines
Year

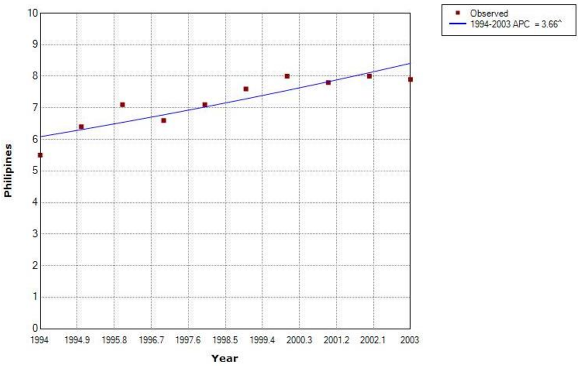
Observed
1994-2003 APC = 3.66^
Philipines
Year

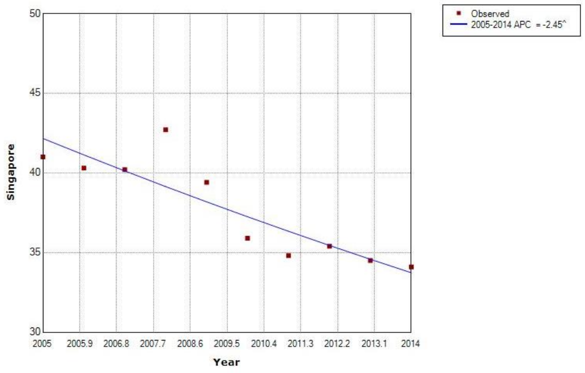

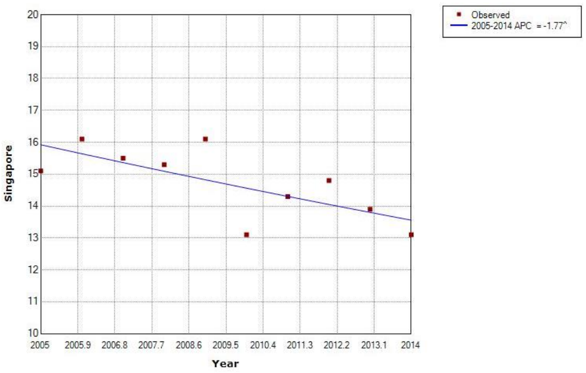

4) Oceania

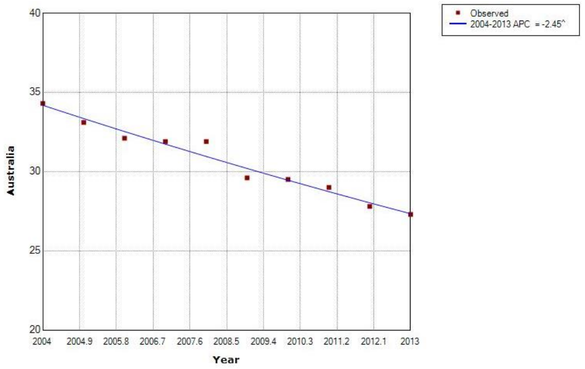

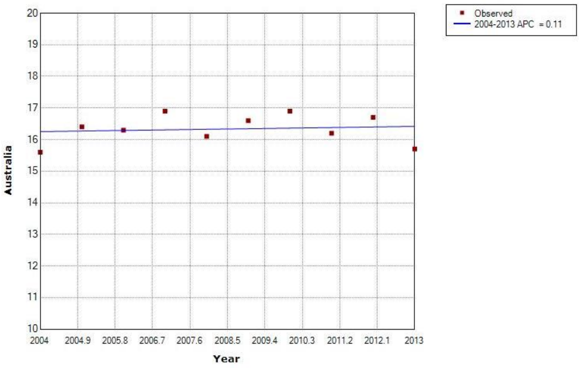

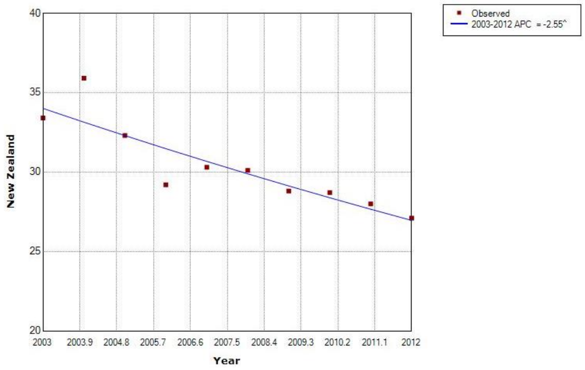

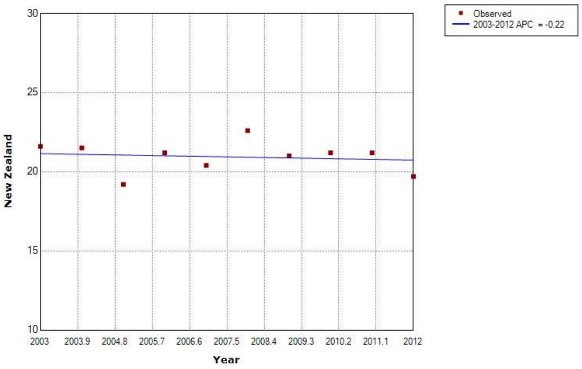

5) Northern Europe

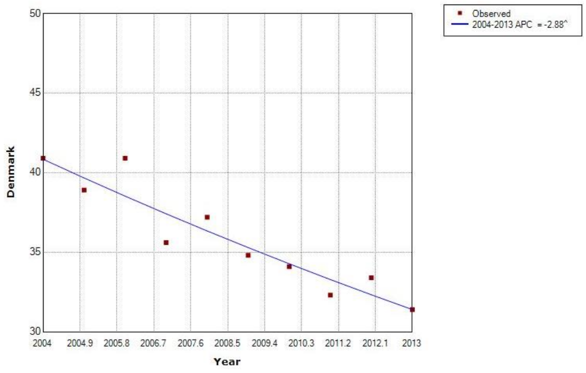

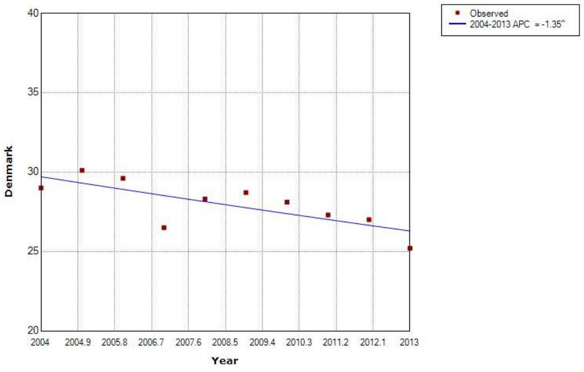

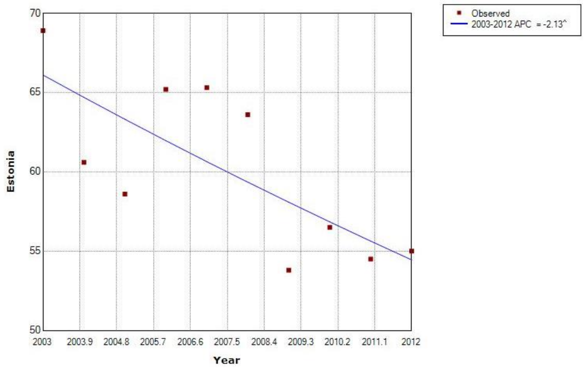
Observed
2003-2012 APC = -2.13^
Estonia
Year
2003 2003.9 2004.8 2005.7 2006.6 2007.5 2008.4 2009.3 2010.2 2011.1 2012
50 55 60 65 70
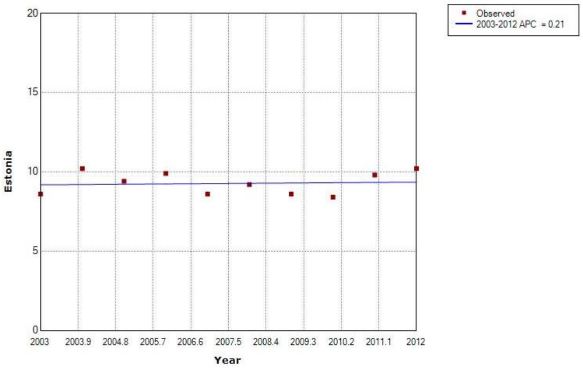
Observed
2003-2012 APC = 0.21
Estonia
Year
2003 2003.9 2004.8 2005.7 2006.6 2007.5 2008.4 2009.3 2010.2 2011.1 2012
0 5 10 15 20
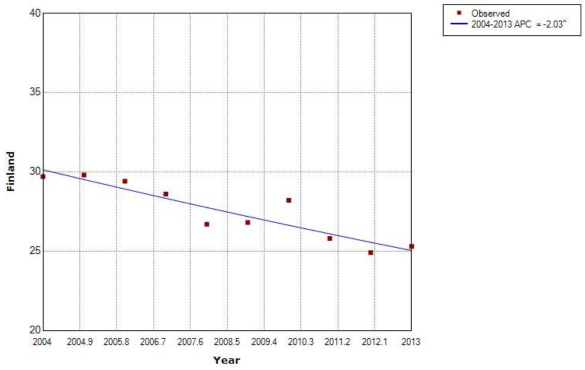
Observed
2004-2013 APC = -2.03^
Finland
Year
2004 2004.9 2005.8 2006.7 2007.6 2008.5 2009.4 2010.3 2011.2 2012.1 2013
20 25 30 35 40
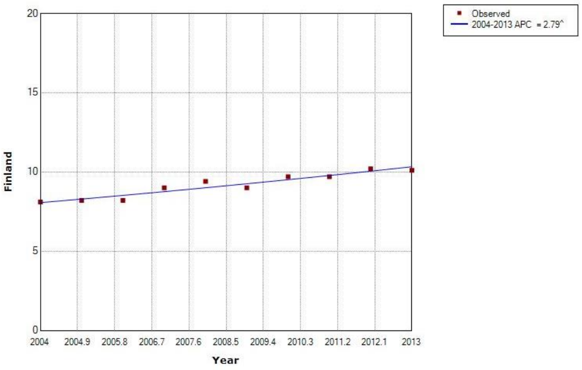
Observed
2004-2013 APC = 2.79^
Finland
Year
2004 2004.9 2005.8 2006.7 2007.6 2008.5 2009.4 2010.3 2011.2 2012.1 2013
0 5 10 15 20
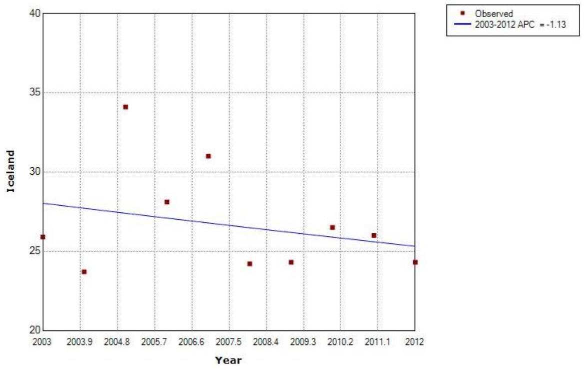
Observed
2003-2012 APC = -1.13
Iceland
Year
2003 2003.9 2004.8 2005.7 2006.6 2007.5 2008.4 2009.3 2010.2 2011.1 2012
20 25 30 35 40
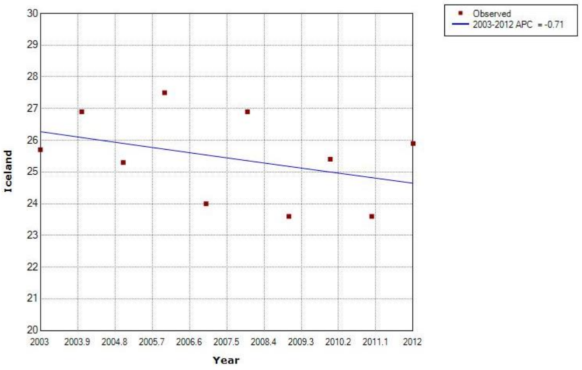
Observed
2003-2012 APC = -0.71
Iceland
Year
2003 2003.9 2004.8 2005.7 2006.6 2007.5 2008.4 2009.3 2010.2 2011.1 2012
20 21 22 23 24 25 26 27 28 29 30
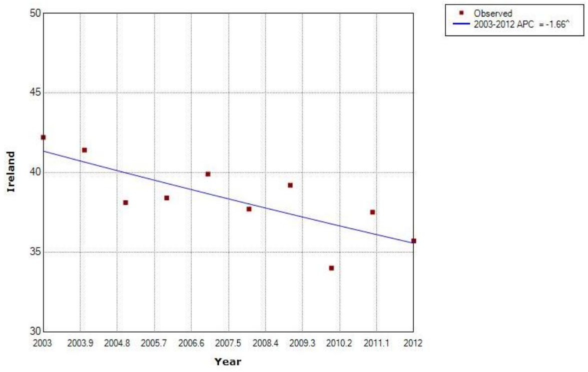
Observed
2003-2012 APC = -1.66^
Ireland
Year
2003 2003.9 2004.8 2005.7 2006.6 2007.5 2008.4 2009.3 2010.2 2011.1 2012
30 35 40 45 50
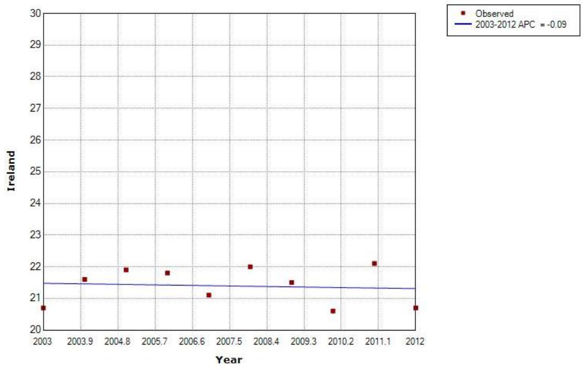
Observed
2003-2012 APC = -0.09
Ireland
Year
2003 2003.9 2004.8 2005.7 2006.6 2007.5 2008.4 2009.3 2010.2 2011.1 2012
20 21 22 23 24 25 26 27 28 29 30
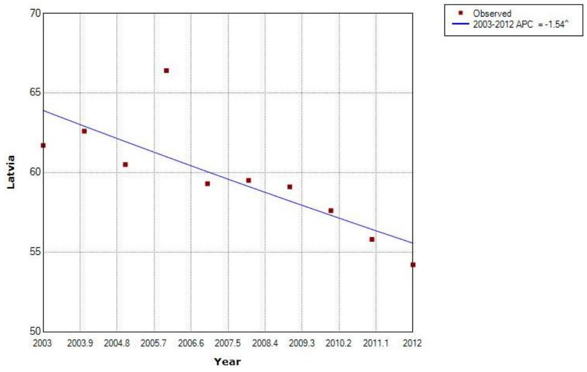
Observed
2003-2012 APC = -1.54^
Latvia
Year
2003 2003.9 2004.8 2005.7 2006.6 2007.5 2008.4 2009.3 2010.2 2011.1 2012
50 55 60 65 70
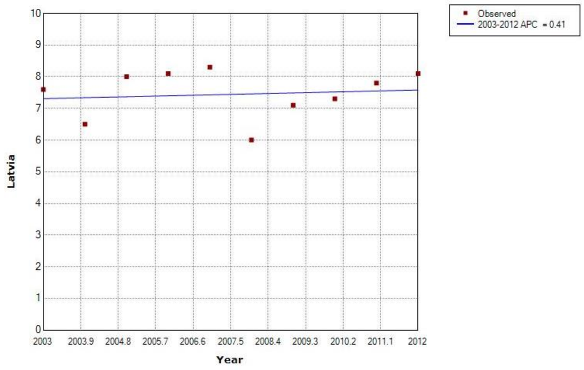
Observed
2003-2012 APC = 0.41
Latvia
Year
2003 2003.9 2004.8 2005.7 2006.6 2007.5 2008.4 2009.3 2010.2 2011.1 2012
0 1 2 3 4 5 6 7 8 9 10

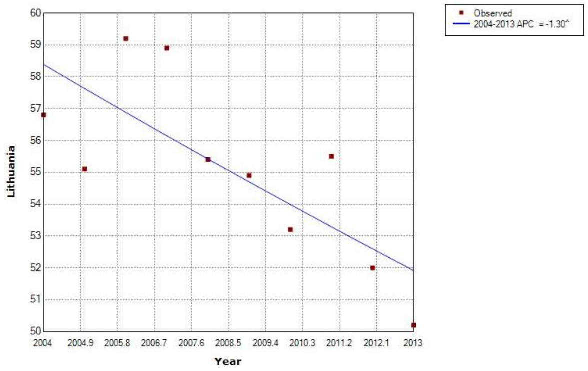
Observed
2004-2013 APC = -1.30^
Lithuania
Year

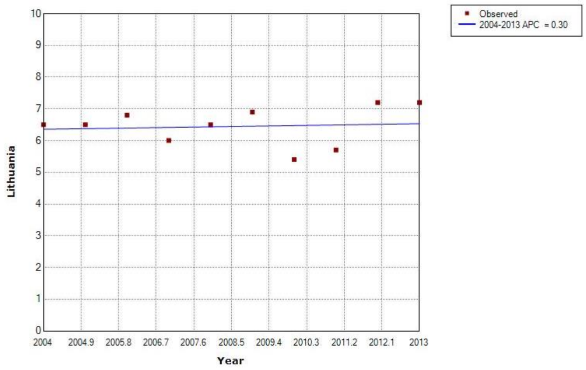
Observed
2004-2013 APC = 0.30
Lithuania
Year

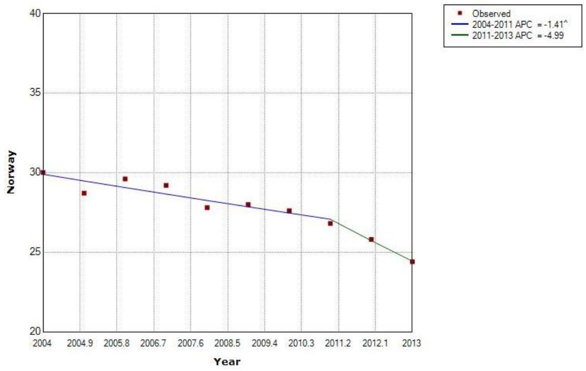
Observed
2004-2011 APC = -1.41^
2011-2013 APC = -4.99
Norway
Year

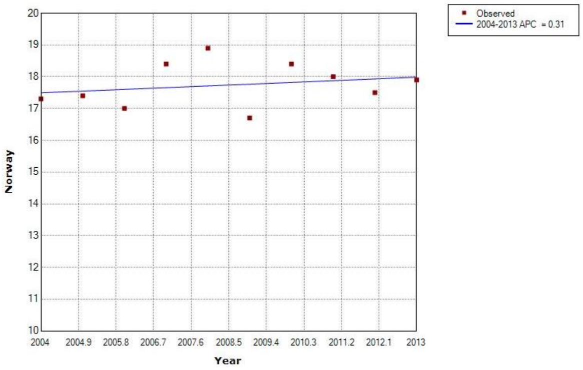
Observed
2004-2013 APC = 0.31
Norway
Year

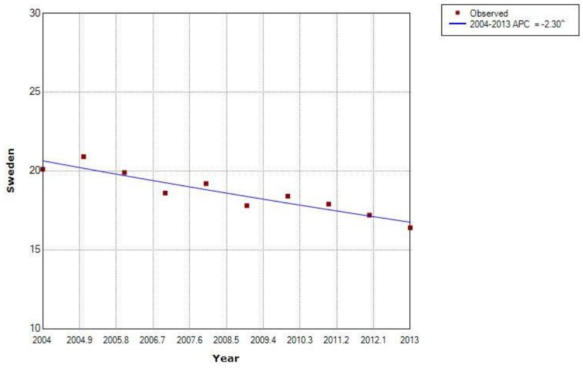
Observed
2004-2013 APC = -2.30^
Sweden
Year

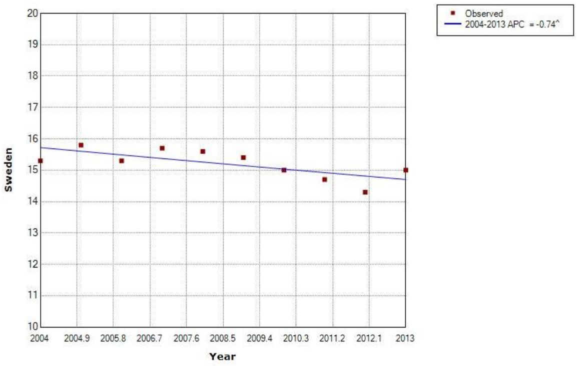
Observed
2004-2013 APC = -0.74^
Sweden
Year

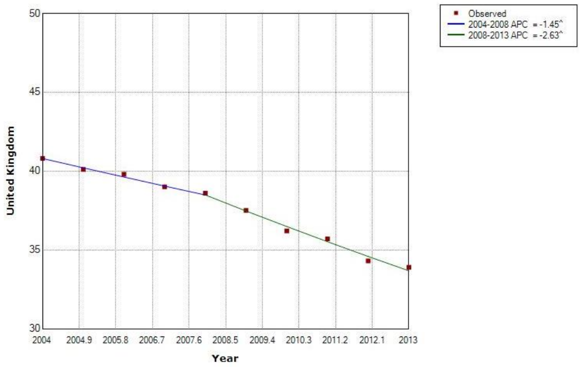
Observed
2004-2008 APC = -1.45^
2008-2013 APC = -2.63^
United Kingdom
Year

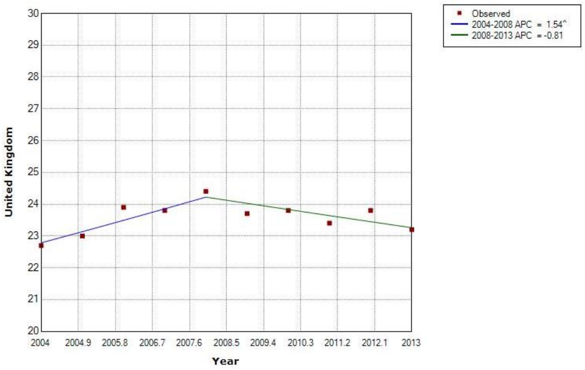
Observed
2004-2008 APC = 1.54^
2008-2013 APC = -0.81
United Kingdom
Year

6) Western Europe

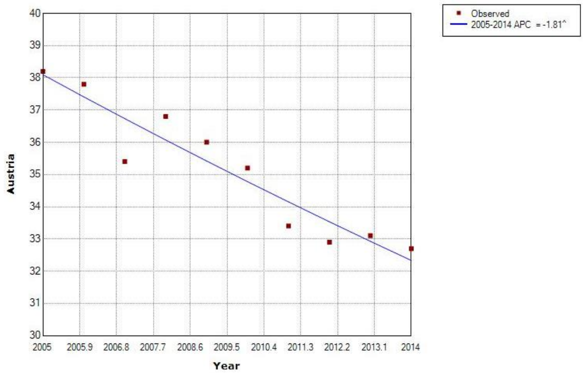

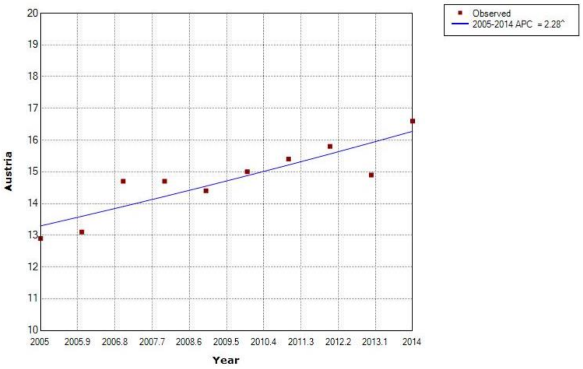

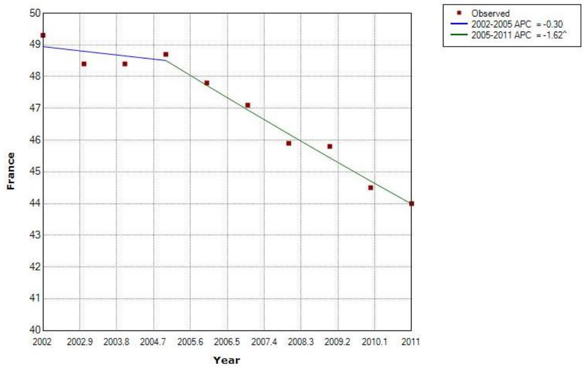

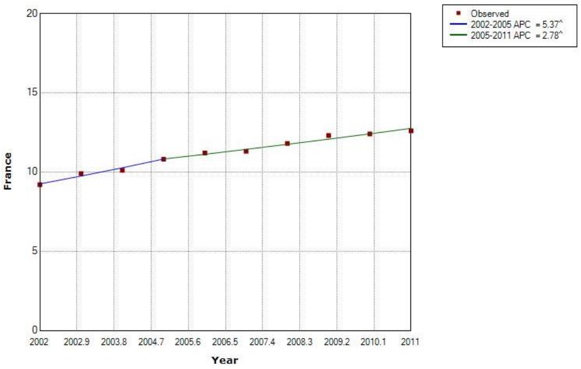

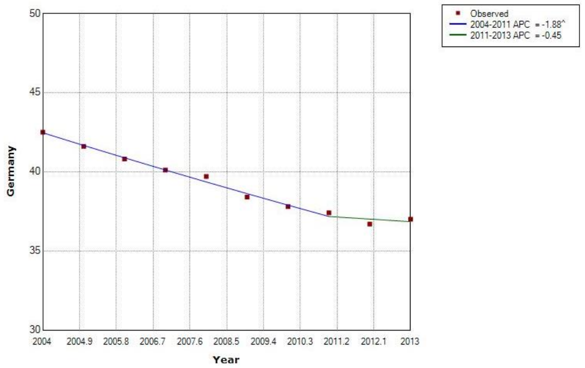

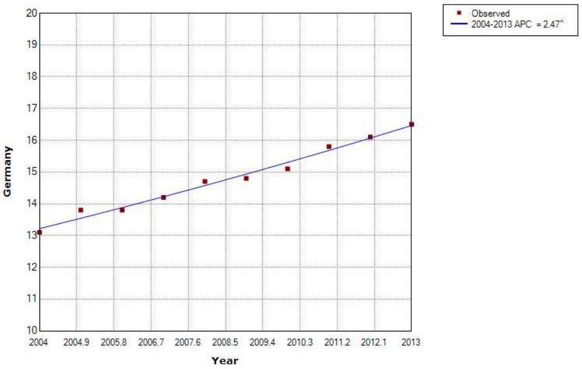

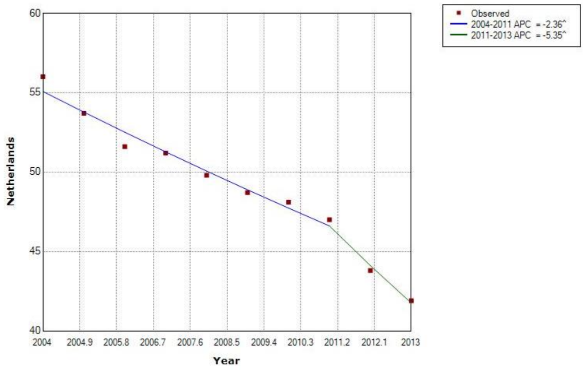

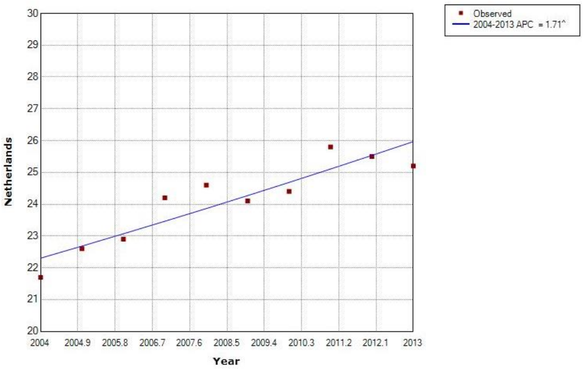

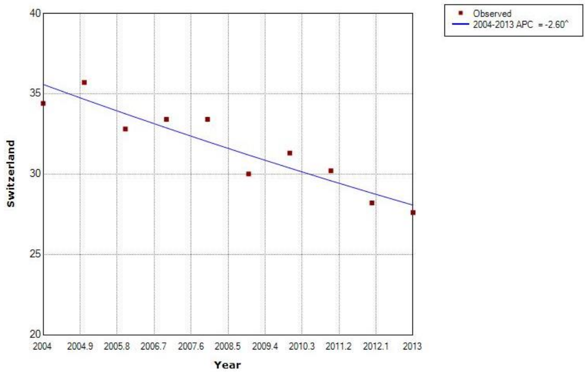

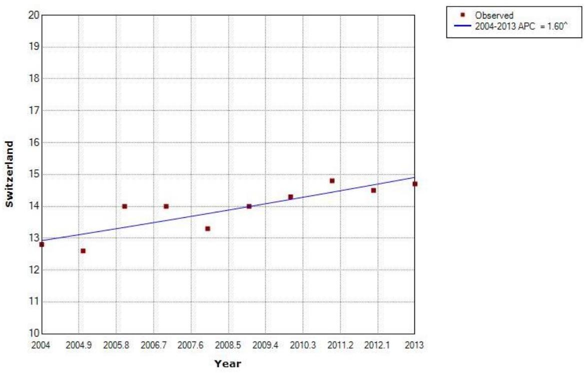

7) Southern Europe

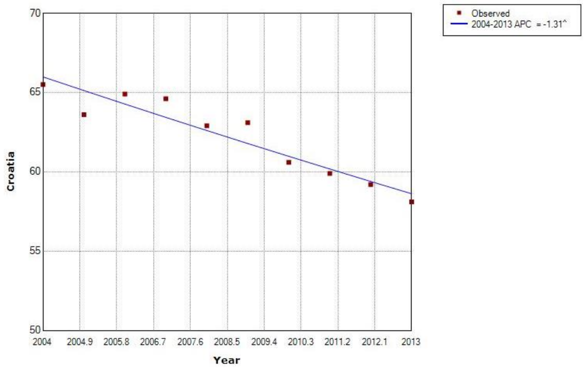

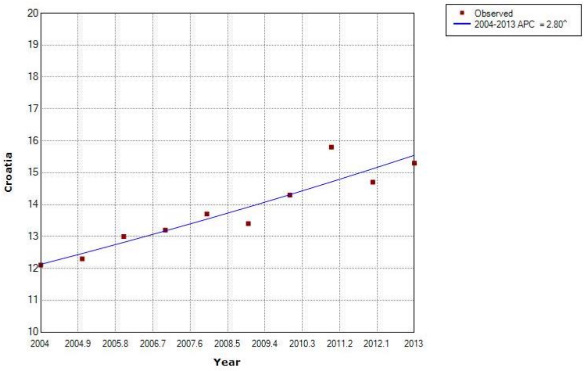

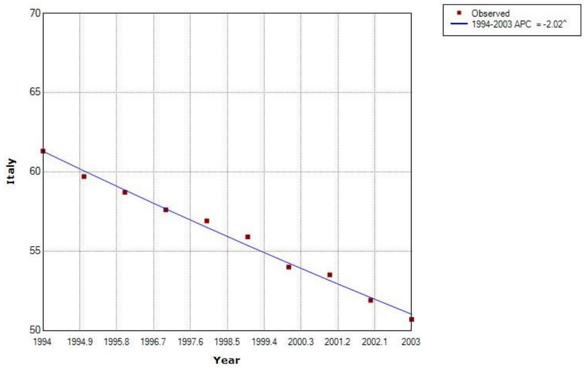

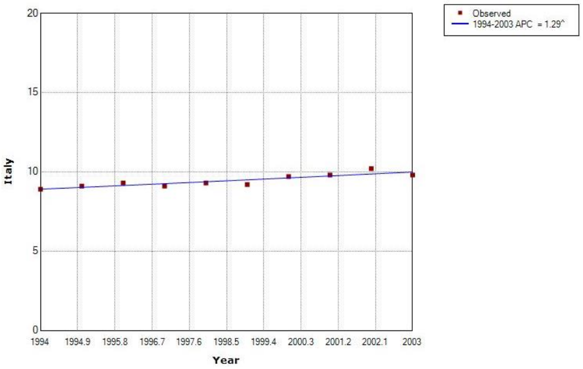

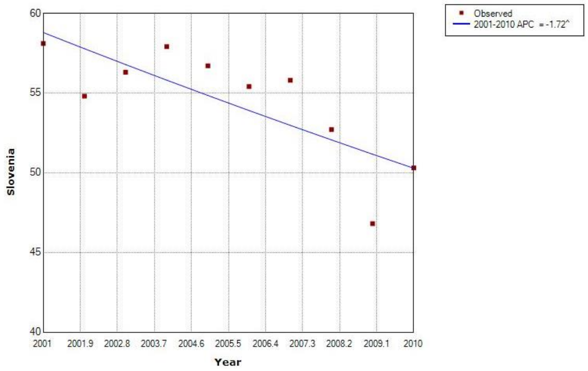

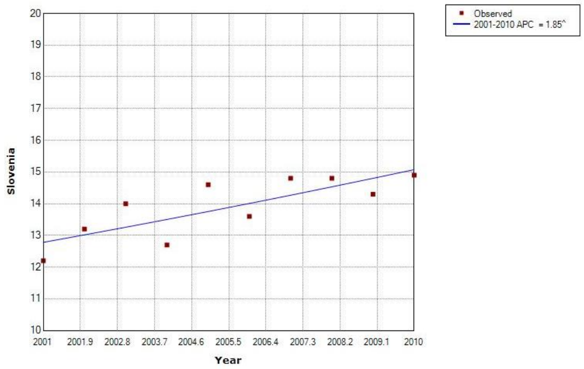

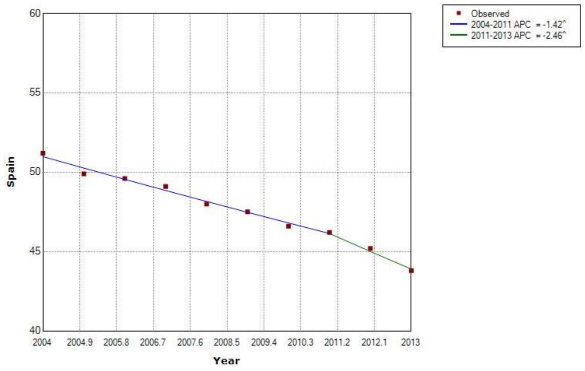

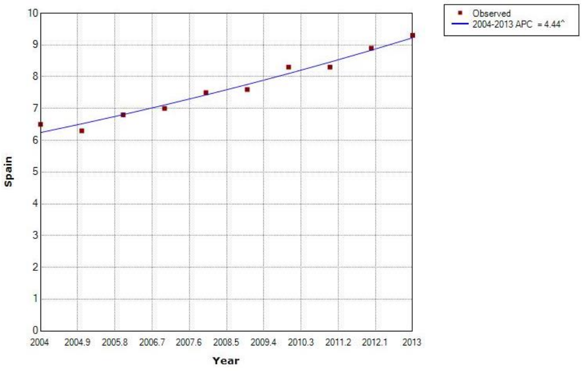

8) Eastern Europe

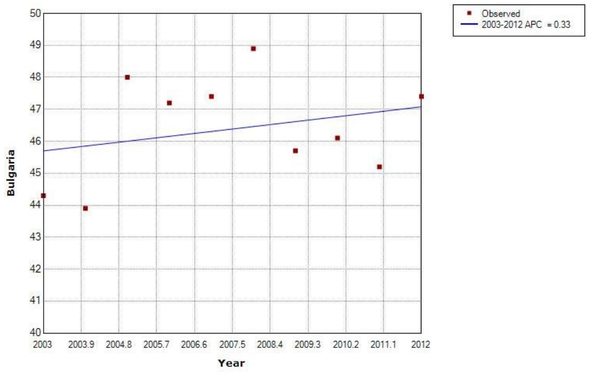

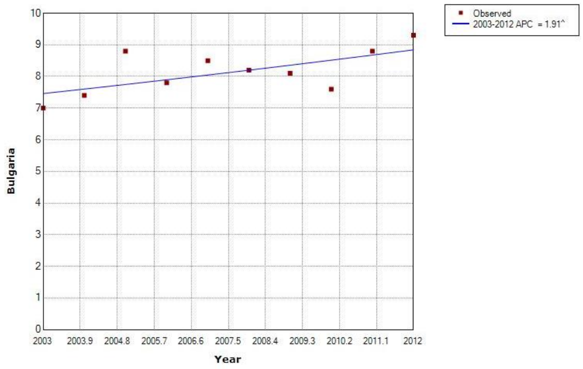

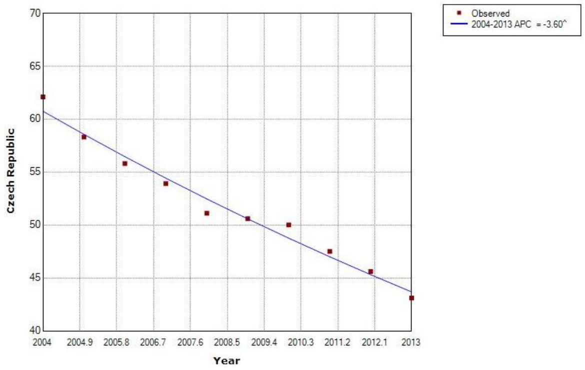

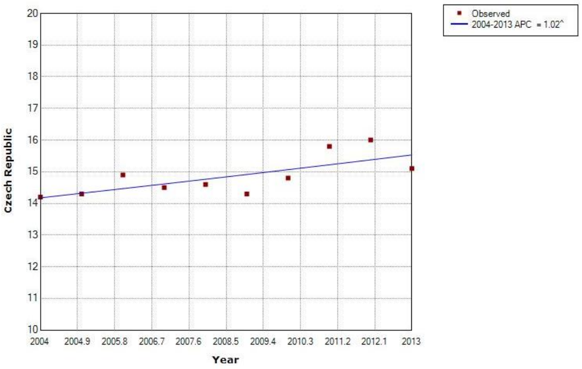

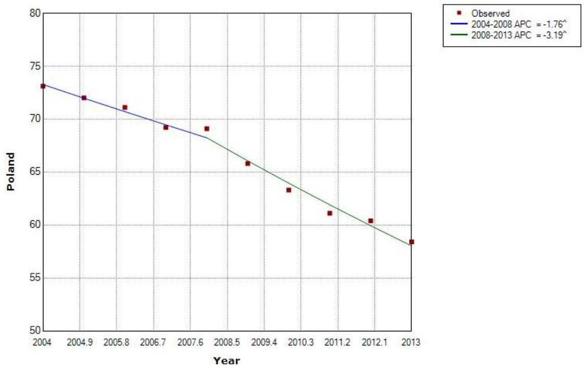

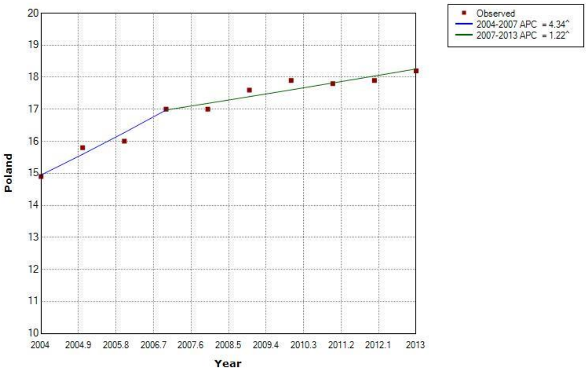

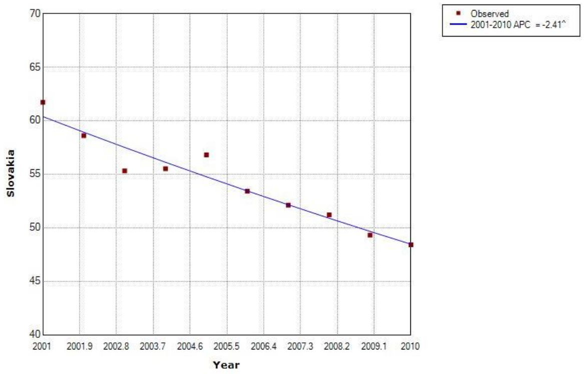

Observed
2001-2010 APC = -2.41^
Slovakia
Year

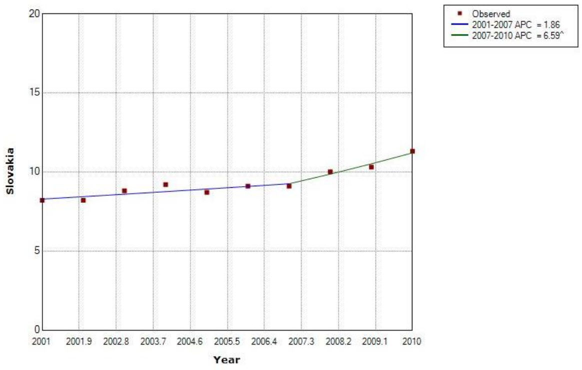

Observed
2001-2007 APC = 1.86
2007-2010 APC = 6.59^
Slovakia
Year
